# High accuracy of genome-enabled prediction of belowground and physiological traits in barley seedlings

**DOI:** 10.1093/g3journal/jkac022

**Published:** 2022-01-31

**Authors:** Damiano Puglisi, Andrea Visioni, Hakan Ozkan, İbrahim Kara, Angela Roberta Lo Piero, Fatima Ezzahra Rachdad, Alessandro Tondelli, Giampiero Valè, Luigi Cattivelli, Agostino Fricano

**Affiliations:** 1 Dipartimento di Agricoltura, Alimentazione e Ambiente (Di3A), Università di Catania, 95123 Catania, Italy; 2 Biodiversity and Crop Improvement Program, International Center for Agricultural Research in the Dry Areas, 6299 Rabat, Morocco; 3 Faculty of Agriculture, Department of Field Crops, University of Cukurova, 01330 Adana, Turkey; 4 Bahri Dagdas International Agricultural Research Institute, Km Karatay/Konya 42020, Turkey; 5 Faculty of Sciences Ben M’sik, Department of Biology, Environment and Ecology Laboratory, Hassan II University of Casablanca, 7955 Casablanca, Morocco; 6 Council for Agricultural Research and Economics—Research Centre for Genomics and Bioinformatics, 29017 Fiorenzuola d’Arda (PC), Italy; 7 DiSIT, Dipartimento di Scienze e Innovazione Tecnologica, Università del Piemonte Orientale, 13100 Vercelli, Italy

**Keywords:** seminal root angle, seminal root number, transpiration rate, MAGIC, barley, genomic prediction, threshold GBLUP, MPP, Multiparental Populations, Multiparent Advanced Generation Inter-Cross (MAGIC)

## Abstract

In plants, the study of belowground traits is gaining momentum due to their importance on yield formation and the uptake of water and nutrients. In several cereal crops, seminal root number and seminal root angle are proxy traits of the root system architecture at the mature stages, which in turn contributes to modulating the uptake of water and nutrients. Along with seminal root number and seminal root angle, experimental evidence indicates that the transpiration rate response to evaporative demand or vapor pressure deficit is a key physiological trait that might be targeted to cope with drought tolerance as the reduction of the water flux to leaves for limiting transpiration rate at high levels of vapor pressure deficit allows to better manage soil moisture. In the present study, we examined the phenotypic diversity of seminal root number, seminal root angle, and transpiration rate at the seedling stage in a panel of 8-way Multiparent Advanced Generation Inter-Crosses lines of winter barley and correlated these traits with grain yield measured in different site-by-season combinations. Second, phenotypic and genotypic data of the Multiparent Advanced Generation Inter-Crosses population were combined to fit and cross-validate different genomic prediction models for these belowground and physiological traits. Genomic prediction models for seminal root number were fitted using threshold and log-normal models, considering these data as ordinal discrete variable and as count data, respectively, while for seminal root angle and transpiration rate, genomic prediction was implemented using models based on extended genomic best linear unbiased predictors. The results presented in this study show that genome-enabled prediction models of seminal root number, seminal root angle, and transpiration rate data have high predictive ability and that the best models investigated in the present study include first-order additive × additive epistatic interaction effects. Our analyses indicate that beyond grain yield, genomic prediction models might be used to predict belowground and physiological traits and pave the way to practical applications for barley improvement.

## Introduction

Despite their importance for the uptake of water and nutrients, belowground traits have been largely neglected for crop improvement as breeding efforts have predominantly targeted aboveground traits related to yield formation. Nevertheless, there is evidence that thousands of years of empirical selection have also indirectly reshaped the root system architecture of domesticated species, corroborating the importance of belowground traits for crop yield and the existence of a correlation between these traits (de Dorlodot *et al.* 2007; Herder *et al.* 2010; Jia et al. 2019). In cereals, experimental results and crop simulation models (CSMs) have pointed out that genotypes with a deeper root system architecture can cope with drought and heat stresses, increasing grain yield (GY) in dry environments (Manschadi *et al.* 2008; Mu *et al.* 2015; Liu *et al.* 2017; Tao *et al.* 2017). For instance, in durum wheat, contrasting root system architectures correlate with drought-intolerant and drought-tolerant genotypes showing higher GY under sub-optimal water regimes (El Hassouni *et al.* 2018). In this species, it has been shown that deeper root system architectures can increase GY from 16% to 35% in environments with limited soil moisture and from 9% to 24% in irrigated sites (El Hassouni *et al.* 2018). Similarly, in bread wheat narrower and deeper root system architectures with more branching at depth allow to provide greater access to soil moisture in environments experiencing terminal drought (Manschadi *et al.* 2008). In maize, it has been shown that the increase of root size improves nitrogen absorption and GY (Mu *et al.* 2015) and that a more efficient root system is more important than canopy architecture for determining plant growth rate and biomass accumulation (Hammer *et al.* 2009). Consequently, the improvement of crops targeting the root system architecture and belowground traits is high desirable to enhance productivity and cope with climate change (Tracy *et al.* 2020).

In cereals, the root system architecture of seedlings can be dissected into primary or seminal roots and nodal or secondary roots. While seminal roots develop first from the primordia of the embryo and grow out from the coleorhizae, the development of nodal roots begins at the tillering stage from the basal nodes of the crown (Wahbi and Gregory 1995). In bread and durum wheat, it has been shown that the seminal root number (SRN) and the seminal root angle (SRA), that is the angle measured between the first pair of seminal roots or between the 2 outmost seminal roots at the seedling stage, are 2 proxy traits that can predict the root system architecture at the adult stages (Manschadi *et al.* 2008; El Hassouni *et al.* 2018; Alahmad *et al.* 2019). For instance, reduced SRA and higher SRN in bread wheat seedlings correlate with drought-tolerant genotypes (e.g. Baxter, Babax, and Dharwar Dry, SeriM82), which exhibit a deeper and more compact root system architecture at the adult stages (Manschadi *et al.* 2008). In barley, the assumption that SRA and SRN measured in seedlings are proxies of the root system architecture of mature plants has not been directly assessed, although in spring, genotypes moderate correlations between these belowground traits and GY have been observed in field trials organized in 20 rainfed and irrigated site-by-season combinations (Robinson *et al.* 2018). Recently, phenotypic variation for SRA and SRN has been assessed in a large panel of spring barley and exploited to map loci that underlie these traits using genome-wide association studies (Jia et al. 2019). Although phenotyping platforms and technologies to analyze the whole root system architecture at mature stage of development are progressively becoming widespread, the incorporation of belowground traits in actual breeding programs is still in its infancy and might benefit from using SRA and SRN, which can be easily scored.

The evaporative demand or vapor pressure deficit (VPD) points out the difference between the saturated and the actual vapor pressure of air at a given temperature and drives the transpiration rate (TR) of crops (Kholová *et al.* 2012). In field conditions, either soil drought or atmospheric drought, that is the combination of high temperatures and low humidity, does not allow crops to satisfy the required evaporative demand and climate change is expected to exacerbate this phenomenon (Lobell and Gourdji 2012; Medina *et al.* 2019). CSMs have pointed out that, beyond the root system architecture, the TR response to VPD is an important physiological trait that might be targeted to cope with high evaporative demand and increase GY (Tao *et al.* 2009, 2017). In fact, the reduction of water flux to leaves for limiting TR at high levels of VPD is a water-saving strategy that imposes physiological trade-offs in leaf dehydration and senescence and allows crops to better manage soil moisture to overcome drought stress. While this water-saving strategy might cause yield penalty when soil moisture is not a limiting factor, in sorghum and maize, experimental evidence has shown that limiting TR at high evaporative demand can allow to increase GY in dry environments (Sinclair *et al.* 2005). As substantiated for belowground traits, TR response to VPD is a key physiological trait that can serve as proxy trait for drought tolerance (Schoppach and Sadok 2012, 2013; Schoppach *et al.* 2016). In durum wheat, the variation of TR response has allowed to identify at least 2 different sets of genotypes showing linear and segmented trends of TR in response to VPD and interestingly, these different responses have been correlated with different GY performances and biomass production in rainfed and irrigated field trials (Medina *et al.* 2019). In sorghum and chickpeas, closing of stomata for limiting TR has been correlated with genotypes that have a better ability to retain soil moisture and contribute to yield formation under drought stress (Devi *et al.* 2015; Sivasakthi *et al.* 2017; Medina *et al.* 2019). While phenotypic diversity in TR response to VPD has been widely assessed in bread and durum wheat (Schoppach and Sadok 2012; Schoppach *et al.* 2017), the knowledge of this trait in barley has lagged behind and to date its natural variation has been assessed in a limited panel of 25 wild barley and in 1 cultivar, which corroborate the existence of untapped diversity for TR in barley germplasm (Sadok and Tamang 2019) that might be exploited for barley improvement and development of more drought-tolerant genotypes.

Genomic prediction (GP) aims to regress genome-wide single nucleotide polymorphisms (SNPs) or other types of DNA markers on phenotypes of individuals to simultaneously predict their effects (Meuwissen *et al.* 2001). The population of individuals having both phenotypic and genotypic information is known as training population (TP) and is used for constructing predictive models, which allow to compute “Genomic Estimated Breeding Values” (GEBVs) in individuals for which only genotyping information is available (Desta and Ortiz 2014). Typically, the predictive models used in GP require to regress a number of predictors (DNA markers) that greatly exceeds the number of observations or phenotypes and several parametric and nonparametric models have been proposed to deal with overfitting and the “large *p*, small *n*” problem (Meuwissen *et al.* 2001; Jannink *et al.* 2010; Pérez and de los Campos 2014b) as in these conditions, the estimation of marker effects using the ordinary least squares method is not practicable. Unlike methods based on whole genome regression of markers, the genomic best linear unbiased prediction (GBLUP) method treats genomic values of individuals as random effects in a linear mixed model and uses a genomic relationship matrix based on DNA marker data to compute GEBVs (VanRaden 2008; Wang *et al.* 2018). Interestingly, under certain assumptions, it has been demonstrated that GBLUP and ridge regression of markers are actually equivalent models (Habier *et al.* 2007). To date, in plant breeding, GP has been mainly applied for improving GY (Crossa *et al.* 2017), but this methodology offers the possibility to predict other traits of agricultural interest that cannot be easily scored (e.g. belowground and physiological traits correlated with abiotic stress tolerances). For instance, GP models have been fitted to seed size (Nielsen *et al.* 2016), thousand grain weight, number of grains per m^2^, grain plumpness (Bhatta *et al.* 2020), root vigor (Biscarini *et al.* 2014), straw breaking and lodging (Tsai *et al.* 2020), beta-glucan and grain protein content (Bhatta *et al.* 2020), and starch (Tsai *et al.* 2020).

In the present study, we examined the diversity and distribution of belowground (SRA and SRN) and physiological (TR response to increasing VPD) traits at the seedling stage in an 8-way Multiparent Advanced Generation Inter-crosses (MAGIC) population of winter barley and in its founder parents (Puglisi *et al.* 2021). We correlated phenotypic data of SRA, SRN, and TR scored in controlled conditions with GY obtained in different site-by-season combinations to re-assess the relevance of belowground and physiological traits of seedlings for the uptake of nutrients and water. Leveraging on phenotypic and genotypic information, we fitted and cross-validated different GP models including different sets of linear predictors and showed that these models can successfully predict SRA, SRN, and TR and might pave the way for incorporating these traits in actual breeding programs, underpinning ideotype breeding and characterizing large plant collections.

## Materials and methods

### Plant materials and genotyping

The MAGIC population examined in this study and used for genome-enabled predictions has been extensively described elsewhere (Puglisi *et al.* 2021). Particularly, the set of 89 MAGIC lines used in the present study (Supplementary Tables 1 and 2) corresponds to the TP previously assembled for fitting multienvironment GP models and genotyped using the barley 50K SNP chip (Puglisi *et al.* 2021). In the present study, 20,426 polymorphic SNPs were used for fitting GP models (Supplementary File 1).

### Phenotyping of plant material

The set of MAGIC lines was phenotyped in the following site-by-season combinations to examine GY and heading date: Fiorenzuola d’Arda (Italy, 2016, 2017, 2018, 2019) at CREA-Centro di Genomica e Bioinformatica (44°55'39.0′′N 9°53'40.6′′E, 78 m above sea level), Marchouch (Morocco, 2016, 2019) at ICARDA Experimental station (33°36'43.5′′N 6°42'53.0′′W, 390 m above sea level), Adana (36°59′52.9′′N 35°20′28.0′′E, 24 m above sea level), and Konya (37°53′37.9′′N 32°37′26.0′′E, 1,005 m above sea level) (Turkey, 2019). These data, excluding phenotypic data collected in Marchouch during the growing season 2018–2019, are part of the data set previously analyzed (Puglisi *et al.* 2021). All these experiments were conducted following local management practices, except for field trials organized in Fiorenzuola d’Arda during 2017–2018 and 2018–2019 growing seasons as they were conducted using 2 different levels of nitrogen fertilization as previously described (Puglisi *et al.* 2021). The detailed procedure for analyzing field trial data and deriving the adjusted means of GY is described in the Supplementary text 1 “Procedure for deriving the adjusted means of grain yield.”

SRA and SRN were phenotyped using the clear pot method (Richard *et al.* 2015; Robinson *et al.* 2016) at ICARDA’s physiology laboratory under controlled temperature and humidity according to the original protocol (Richard *et al.* 2015) and using transparent ANOVApot pots (Anovapot Pty Ltd, Brisbane, QLD, Australia, www.anovapot.com/php/anovapot.php) with a diameter of 200 mm, height of 190 mm, and a volume of 4 L. The detailed procedure and the experimental design to phenotype SRA and SRN and for computing the adjusted means of SRA is described in the Supplementary text 2 “Procedure for phenotyping SRA and SRN and deriving the adjusted means of SRA.”

The TR response under progressive VPD was examined at ICARDA’s physiology laboratory under controlled conditions. This experiment was designed randomizing the 90 MAGIC lines using 3 biological replicates per genotype, using 2 L pots with diameter and height of 104 and 200 mm, respectively. In each pot, plants were sown at a depth of circa 2 cm and were uniformly irrigated every 2 days. At Zadoks stage 14 (Zadoks *et al.* 1974), which was reached after 4–5 weeks after sowing, depending on the genotype, pots were irrigated until reaching the maximum water holding capacity of the substrate. The day after, pots were subsequently closed with plastic bags and balls in order to limit evaporation. TR was measured under increasing VPD ranging from 0.4 to 5.4 kPa in a greenhouse under controlled conditions (temperature and humidity) accurately monitored with data loggers (Type TGU-4550, Gemini Data Loggers, UK). Phenotyping of plants and the computation of TR under increasing VPD conditions was carried out following published protocols (Fletcher *et al.* 2007; Sadok and Sinclair 2009a, 2009b; Schoppach and Sadok 2012; Schoppach *et al.* 2017; Tamang and Sadok 2018; Sadok and Tamang 2019) and described in detail in the Supplementary text 3 “Procedure for estimating the TR at different values of VPD.”

### Descriptive statistics and correlation analyses

Variation in GY and SRA in the panel MAGIC lines was investigated computing maximum and minimum values, mean, median, and standard deviation (SD). This descriptive analysis was computed using “metan” package (Olivoto and Lúcio 2020) implemented in R 4.0.3 statistical (R Core Team 2019). The adjusted means of GY and SRA, along with SRN and TR measured at a VPD of 2.7 kPa were analyzed and correlated each other. Two different types of correlations were applied on the basis of variable type: Pearson’s correlation coefficient was applied to compute correlation between continuous traits (GY, SRA, and TR measured at a VPD of 2.7 kPa), while polyserial correlations were computed to measure correlations between continuous and categorical variables (Drasgow 2006). These latter set of correlation analyses was computed using “polycor” package (Fox 2010) implemented in R 4.0.3 statistical (R Core Team 2019).

### GP models fitted to SRN

In the present study, 2 different GP models were fitted to SRN combining phenotypic data with genotypic information obtained with the Barley 50 k SNP chip (Puglisi *et al.* 2021). For this trait, GP models were fitted following 2 different hypotheses. First, we assumed that SRN varies as an ordinal discrete variable that indicates the performance of plants at the adult stage under nitrogen or water deficiency and for this aim, threshold genomic best linear unbiased predictor (TGBLUP) models and extended TGBLUP models were fitted.

Formal presentation of the model theory of GP for ordinal discrete data was disserted elsewhere (Montesinos-López *et al.* 2015a). Here, we shortly introduce the TGBLUP models used in the present study for implementing GP. For SRN, we assumed that the ordinal response variable $y_{ik}$, that is the number of observed seminal roots, can take *C* =7 mutually exclusive c values, where i indicates the genotype, k points out the number of replicates, and c takes values equal to the number of observed seminal roots observed in the MAGIC population, that is c=2, 3, 4, 5, 6, 7, 8. Moreover, we supposed that the ordinal response variable $y_{ik}$ follows a multinomial distribution with parameters $N_{ik}$ and $\pi_{ik\left(c=2\right)},\;\pi_{ik\left(c=3\right)},\;\pi_{ik\left(c=4\right)},\;\pi_{ik\left(c=5\right)},\;\pi_{ik\left(c=6\right)},\;\pi_{ik\left(c=7\right)},\;\pi_{ik\left(c=8\right)}$, that is ($y_{ik\left(c=2\right)},\;y_{ik\left(c=3\right)},\;y_{ik\left(c=4\right)},\;y_{ik(c=5)},\;y_{ik(c=6)},\;y_{ik(c=7)},\;y_{ik(c=8)})∼\mathrm{MULTINOMIAL}(N_{ik},\;\pi_{ik\left(c=2\right)},\;\pi_{ik\left(c=3\right)},\;\pi_{ik\left(c=4\right)},\;\pi_{ik\left(c=5\right)},\;\pi_{ik\left(c=6\right)},\;\pi_{ik\left(c=7\right)},\;\pi_{ik\left(c=8\right)})$

where $N_{ik}$ points out the number of observation and $\pi_{ik\left(c=2\right)},\;\pi_{ik\left(c=3\right)},\;\ldots \pi_{ik\left(c=8\right)}$ point out the probabilities of getting values c=2, 3, …8 in the $i^{th}$ genotype in the $k^{th}$ replicate. Threshold models assume that $y_{ik}$ is generated from an underlying continuous random variable $l_{ik}$, having a normal distribution, which is called latent “liability” variable (Sorensen *et al.* 1995; Montesinos-López *et al.* 2015a) and imply that for C ordinal and mutually exclusive categories the existence of C-1=6 unknown γ thresholds that must be estimated such as $\gamma_{\min}{<\gamma }_{1}<\gamma_{2}<\gamma_{3}.{<\gamma }_{\max}$, with $\gamma_{\min}=-\infty$ and $\gamma_{\max}=+\infty$. In threshold models, values of $l_{ik}$ are mapped to the ordinal categorical response according to the following conditions:

yik=2 if γmin<lik<γ1 3 if γ1<lik<γ2 4 if γ2<lik<γ3 .…..………………8 if γ6<lik<γmax .

In these models, the link function relating linear predictors with the probability of observing data is the cumulative probit Φ(.), that is the cumulative distribution function of a standard normal distribution and $\Phi^{-1}$ is the corresponding inverse function. Consequently, threshold models are specified with C-1 linear predictors $\eta_{\mathrm{ikc}}$ as follows:

$$\begin{array}{c} \eta_{ik\left(c=2\right)}=\Phi^{-1}\left(\pi_{ik\left(c=2\right)}\right)=\gamma_{1}-X_{ik}^{T}\beta -Z_{ik}^{T}u\\ \eta_{ik\left(c=3\right)}=\Phi^{-1}\left(\pi_{ik\left(c=2\right)}+\pi_{ik\left(c=3\right)}\right)=\gamma_{2}-X_{ik}^{T}\beta -Z_{ik}^{T}u\\ \eta_{ik\left(c=4\right)}=\Phi^{-1}\left(\pi_{ik\left(c=2\right)}+\pi_{ik\left(c=3\right)}+\pi_{ik\left(c=4\right)}\right)=\gamma_{3}-X_{ik}^{T}\beta -Z_{ik}^{T}u\\ \eta_{ik\left(c=5\right)}=\Phi^{-1}\left(\pi_{ik\left(c=2\right)}+\pi_{ik\left(c=3\right)}+\pi_{ik\left(c=4\right)}+\pi_{ik\left(c=5\right)}\right)\\ =\gamma_{4}-X_{ik}^{T}\beta -Z_{ik}^{T}u\\ \eta_{ik\left(c=6\right)}=\Phi^{-1}\left(\pi_{ik\left(c=2\right)}+\pi_{ik\left(c=3\right)}+\pi_{ik\left(c=4\right)}+\pi_{ik\left(c=5\right)}+\pi_{ik\left(c=6\right)}\right)\\ =\gamma_{5}-X_{ik}^{T}\beta -Z_{ik}^{T}u\\ \eta_{ik\left(c=7\right)}=\Phi^{-1}\left(\pi_{ik\left(c=2\right)}+\pi_{ik\left(c=3\right)}+\pi_{ik\left(c=4\right)}+\pi_{ik\left(c=5\right)}+\pi_{ik\left(c=6\right)}+\pi_{ik\left(c=7\right)}\right)\\ =\gamma_{6}-X_{ik}^{T}\beta -Z_{ik}^{T}u \end{array}$$

where $X_{ik}^{T}$ is a known row incidence vectors of fixed effects, $Z_{ik}^{T}$ is a known row incidence vectors of random effects, β points out the vector of fixed effects, and b is the vector of random effects. The probabilities $\pi_{\mathrm{ikc}}$ are linked to the linear predictors $\eta_{\mathrm{ikc}}$ as follows:

$$\begin{array}{c} \pi_{ik\left(c=2\right)}=\Phi \left(\pi_{ik\left(c=2\right)}\right)\\ \pi_{ik\left(c=3\right)}+\pi_{ik\left(c=2\right)}=\Phi \left(\pi_{ik\left(c=3\right)}\right)\\ \ldots ..\\ \pi_{ik(c=7)}+\pi_{ik(c=6)}+\pi_{ik(c=5)}+\pi_{ik(c=4)}+\pi_{ik(c=3)}+\pi_{ik(c=2)}=\Phi \left(\pi_{ik\left(c=7\right)}\right)\;. \end{array}$$

As mentioned above, threshold models assume that the latent and normally distributed variable $l_{ik}$ generates the observed C categories as follows:

(1) $$l_{ik}=X_{ik}^{T}\beta +Z_{ik}^{T}u+\;e_{ik}$$

where the error terms $e_{ik}$ are independent and identically distributed and follow a normal distribution with mean 0 and SD equals to 1, that is $e_{ik}\;∼N(0,1)$. In the present study, different combinations of linear predictors, including replicates, lines, markers, and first-order epistatic effects, were incorporated in $X_{ik}^{T}$ and $Z_{ik}^{T}$ for fitting 5 extended threshold models (Table 1), which were already substantiated and described in other studies (Jarquín *et al.* 2014; Montesinos-López *et al.* 2015a).

**Table 1.** Summary of the linear predictors incorporated in the GBLUP and TGBLUP models used to analyze SRN, SRA, and TR.

Model	Main effects			Interaction
	R	L	G	G × G
SRN-Model 1 SRN-log-Model 1 SRA-Model 1 TR-Model 1	×	×		
SRN-Model 2 SRN-log-Model 2 SRA-Model 2 TR-Model 2	×		×	
SRN-Model 3 SRN-log-Model 3 SRA-Model 3 TR-Model 3	×		×	×
SRN-Model 4 SRN-log-Model 4 SRA-Model 4 TR-Model 4	×	×	×	
SRN-Model 5 SRN-log-Model 5 SRA-Model 5 TR-Model 5	×	×	×	×

G, marker covariates; G × G, first-order additive × additive epistasis; L, line; R, replicate.

The resulting 5 models include the following sets of linear predictors:

(2) $$\mathrm{SRN}-\mathrm{Model}\;1:\;l_{ik}=R_{k}\;+\;L_{i}\;+\;\varepsilon_{ik}$$

(3) $$\mathrm{SRN}-\mathrm{Model}\;2:\;l_{ik}={\;R}_{k}\;+\;g_{i}\;+\;\varepsilon_{ik}$$

(4) $$\mathrm{SRN}-\mathrm{Model}\;3:l_{ik}=\;R_{k}\;+\;g_{i}\;+g_{Ai}\;+\;\varepsilon_{ik}$$

(5) $$\mathrm{SRN}-\mathrm{Model}\;4:l_{ik}=\;R_{k}\;+\;L_{i}\;+\;g_{i}+\;\varepsilon_{ik}$$

(6) $$\mathrm{SRN}-\mathrm{Model}\;5:l_{ik}=\;R_{k}\;+\;L_{i}\;+\;g_{i}+g_{Ai}+\;\varepsilon_{ik}$$

where $l_{ik}$ is the latent “liability” variable of $k^{th}$ replicates in the $i^{th}$ line. SRN-Model 1 includes $R_{k}$, which is the fixed effect of *k^th^* replicates and $L_{i}$ that is the random effect of the $i^{th}$ line supposed to be independent and normally distributed as $L_{i}∼N\left(0,\sigma_{L}^{2}\right)$. SRN-Model 2 includes $g_{i}$, which points out the additive genetic value of the $i^{th}$ line, that is $g_{i}=\sum_{n=1}^{p}x_{in}b_{n}$, where $x_{in}$ is the genotype of the $i^{th}$ line at marker n and $b_{n}$ is the corresponding effect of marker n. The vector of additive genetic value $g=(g_{1},\;g_{2},\;g_{3}\ldots \ldots g_{i})$ is supposed to be normally distributed as $g_{\;}∼N\left(0,{G\sigma }_{g}^{2}\right)$ with mean 0 and variance–covariance structure ${G\sigma }_{g}^{2}$, where $\sigma_{g}^{2}$ points out the additive genetic variance $\sigma_{g}^{2}$ and **G** is the genomic marker relationship matrix (VanRaden 2008). SRN-Model 3 extends SRN-Model 2 including first-order multiplicative epistatic effects $g_{A}=(g_{A1},\;g_{A2},\ldots g_{Ai})$, which are assumed to be distributed as $g_{A}∼N\left(0,G_{A}\sigma_{g_{A}}^{2}\right)$, that is the vector of epistatic effects follows a normal distribution with mean 0 and epistatic additive × additive genetic variance $\sigma_{gA}^{2}$ (Montesinos-López *et al.* 2015a). Finally, SRN-Model 4 includes $R_{k},L_{i},\;\mathrm{and}\;g_{i}$ as linear predictors, while SRN-Model 5 extends SRN-Model 4 including $g_{Ai}$ effects. In the present study, the aforementioned threshold models were implemented in a Bayesian framework using BGLR package (Pérez and De Los Campos 2014a) in R 4.0.3 statistical (R Core Team 2019) using default prior distributions and modifying codes published in other studies (Montesinos-López *et al.* 2015a).

Second, we handled SRN as count data for predicting this trait *per se*, fitting 5 log-normal GP models based on GBLUP and indicated as SRN-log-Model 1–5 (Table 1) (Montesinos-López *et al.* 2015b). In SRN-log-Model 1–5, the response variable is the logarithm of SRN, that is ${\log(y}_{kj}+1)$, and was fitted using the same sets of linear predictors ($R_{k}$, $L_{j}$,$\;g_{j}$, $g_{Aj}$) described for the 5 extended TGBLUP models [Table 1; Equations (2–6)]. In these models, $R_{k}$, $L_{j}$,$\;g_{j}$, $g_{Aj}$ follow the same distributions defined for the extended TGBLUP models except for the error terms $\varepsilon_{\mathrm{ik}}$ of $K^{th}$ replicates in $i^{th}$ line, which in these models is distributed as $\varepsilon_{ik}∼N\left(0,\sigma_{e}^{2}\right)$, that is the residuals are independent and normally distributed with mean 0 and variance $\sigma_{e}^{2}$. Like TGBLUP models, log-normal models were implemented using BGLR package (Pérez and De Los Campos 2014b) in R 4.0.3 statistical (R Core Team 2019).

### GP models fitted for SRA and TR

The 5 sets of linear predictors used in the extended TGBLUP models (Table 1) were used for predicting SRA and TR measured at a VPD of 2.7 kPa, using the following models:

(7) $$\mathrm{SRA}-\mathrm{Model}\;1:\;y_{i}=L_{i}+\varepsilon_{i}$$

(8) $$\mathrm{SRA}-\mathrm{Model}\;2:\;y_{i}=g_{i}+\varepsilon_{i}$$

(9) $$\mathrm{SRA}-\mathrm{Model}\;3:\;y_{i}\;=g_{i}\;+g_{Ai}\;+\;\varepsilon_{i}$$

(10) $$\mathrm{SRA}-\mathrm{Model}\;4:\;y_{i}\;=L_{i}\;+\;g_{i}+\;\varepsilon_{i}$$

(11) $$\mathrm{SRA}-\mathrm{Model}\;5:\;y_{i}\;=L_{i}\;+\;g_{i}+g_{Ai}+\;\varepsilon_{i}$$

(12) $$\mathrm{TR}-\mathrm{Model}\;1:\;y_{i}=L_{i}+\varepsilon_{i}$$

(13) $$\mathrm{TR}-\mathrm{Model}\;2:\;y_{i}=g_{i}+\varepsilon_{i}$$

(14) $$\mathrm{TR}-\mathrm{Model}\;3:\;y_{i}\;=g_{i}\;+g_{Ai}\;+\;\varepsilon_{i}$$

(15) $$\mathrm{TR}-\mathrm{Model}\;4:\;y_{i}\;=L_{i}\;+\;g_{i}+\;\varepsilon_{i}$$

(16) $$\mathrm{TR}-\mathrm{Model}\;5:\;y_{i}\;=L_{i}\;+\;g_{i}+g_{Ai}+\;\varepsilon_{i}$$

where $y_{i}$ is the adjusted mean of SRA [Equations (7–11)] or TR [Equations (12–16)], $\epsilon_{i}$ is the error term of the $i^{th}$ measurement with $\epsilon_{i}\;∼N\left(0,\sigma_{\varepsilon }^{2}\right)$, that is that the errors are independent and identically distributed with mean 0 and variance $\sigma_{\varepsilon }^{2}$. In these models, the linear predictors $L_{j}$,$\;g_{j}$, and $g_{Aj}$ follow the same distribution defined for TGBLUP models. These extended GBLUP models (SRA-Models 1–5, TR-Model 1–5) were implemented using BGLR package (Pérez and De Los Campos 2014a) in R 4.0.3 statistical (R Core Team 2019) as censored data described with the following interval

$$a_{i}<y_{i}<b_{i}\;,$$

where $y_{i}$ is the adjusted mean of SRA or TR computed as best linear unbiased estimator (BLUE), $a_{i}$ is the lower bound estimate of $y_{i}$ computed as the difference between $y_{i}$ and 2 SD, and $b_{i}$ is the upper bound estimate of $y_{i}$ computed as the sum of $y_{i}$ with 2 SD.

### Cross-validation of GP models

For the extended TGBLUP models, leave-one-out (LOO) cross-validation was carried out and predictive ability was estimated using both Brier Score (BS) and the proportion of cases correctly classified (PCCC) (Brier 1950; Montesinos-López *et al.* 2015a, 2020). BS was computed as follows:

(17) $$\mathrm{BS}\;=n^{-1}\sum_{i=1}^{n}\;\sum_{c=1}^{g}\;\left({\hat{\pi }}_{\mathrm{ic}}-d_{\mathrm{ic}}\right){\;}^{2}\;,$$

where $\left({\hat{\pi }}_{\mathrm{ic}}-d_{\mathrm{ic}}\right){\;}^{2}$ is the average square difference between ${\hat{\pi }}_{ic}$ predictions and $d_{ic}$ classes for observation *i* into category *c*. BS obtained with Equation (17) was divided by 2 in order to have a range that varies from 0 to 1 (Brier 1950; Montesinos-López *et al.* 2015a). For the other models used in the present study (extended GBLUP and log-normal models), the predictive accuracy of GP models was calculated as the Pearson’s correlation coefficient between GEBVs and the corresponding adjusted means of the trait (SRA, TR measured at a VPD of 2.7 kPa). Unlike the Pearson’s correlation coefficient used for the extended GBLUP models for SRA and TR, lower values of BS point out higher predictive ability of the models, while higher values of BS point out lower predictive ability of models.

## Results

### Phenotypic distribution and analysis of belowground and physiological traits in the barley MAGIC population

**Fig. 1.**
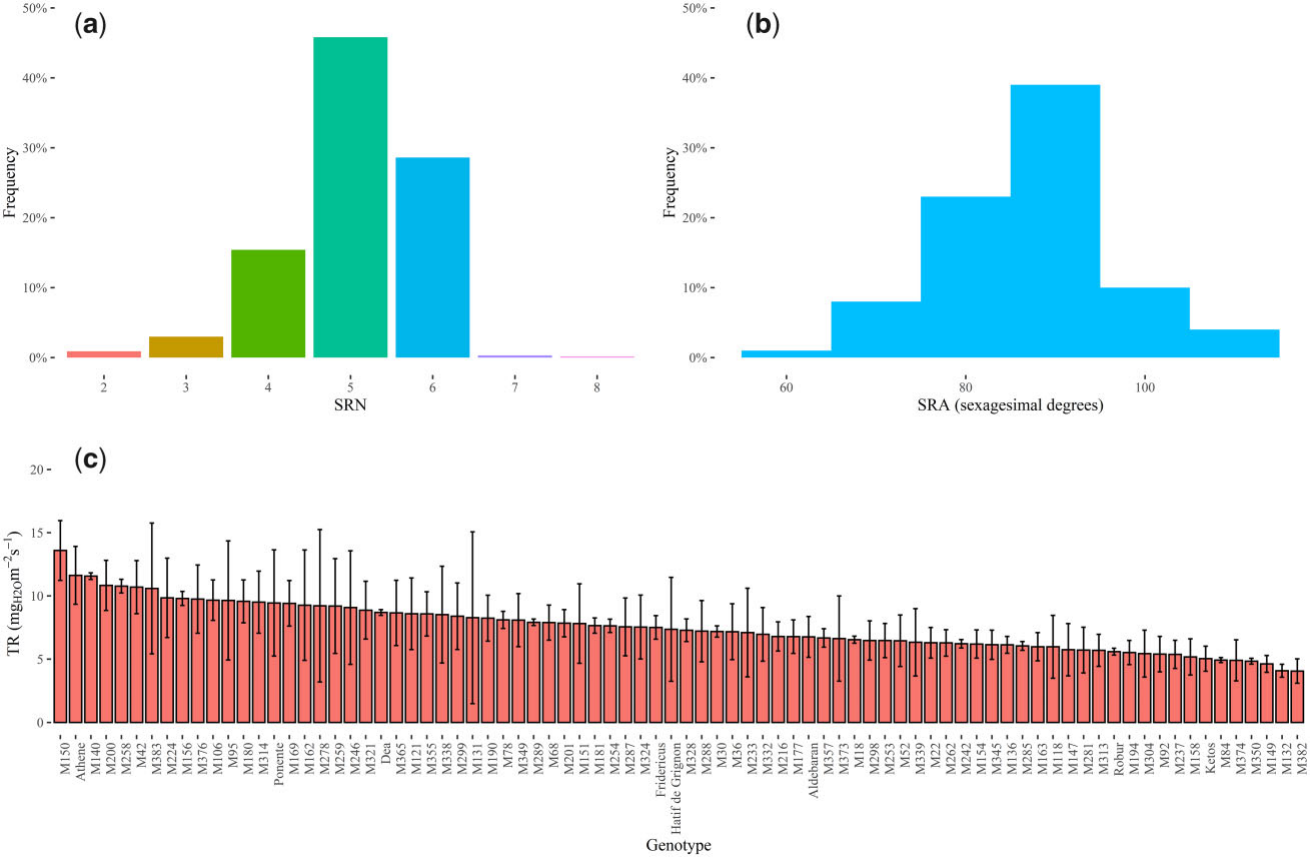
Phenotypic distribution of SRA, SRN, and whole-plant TR measured at a VPD of 2.7 kPa in the panel of MAGIC lines: a) histogram of SRN counted in the MAGIC lines; b) histogram of the adjusted means of SRA measured in sexagesimal degrees; c) bar plot of TR measured at a VPD of 2.7 kPa. Error bars point out the 95% confidence interval of TR values.

To assess the variability of SRA and SRN, the panel of MAGIC lines was phenotyped at the seedling stage under controlled conditions. This analysis showed that SRN varies greatly in the MAGIC population as it ranges between 2 and 8 with a mean of 5 seminal roots and a SD of 0.84 (Fig. 1a; Supplementary Table 1). The adjusted means of SRA and the corresponding 95% confidence intervals of estimates were computed using BLUEs, analyzing the adopted experimental design with a linear mixed model, which was fitted to the raw measurements of SRA. This analysis indicated that the phenotypic distribution of SRA ranges from 57.75° (genotype “M18”) to 106.40° (genotype “M324”) with an average value of 86.61° (Fig. 1b; Supplementary Table 2). Both belowground traits exhibit a bell-shaped distribution (Fig. 1, a and b) and particularly, Shapiro–Wilk normality test showed that, for SRA, the null hypothesis, that is that the adjusted means of SRA follow a normal distribution, cannot be rejected (*P-*value = 0.1844).

The whole-plant TR was measured in the set of MAGIC lines at the seedling stage using increasing VPD values ranging from 0.4 to 5.4 kPa. Regression of whole-plant TR on VPD values was carried out using linear and segmented models and *R*-squared was used as goodness-of-fit measure for model selection. Regression of whole-plant TR on VPD values showed a segmented response in a large fraction of genotypes, while in 28 MAGIC lines model fitting and selection indicated a linear response of TR to increasing levels of VPD (Supplementary Table 2). The breakpoint values of genotypes showing a segmented trend of TR to increasing VPD ranged from 2.3 to circa 2.5 kPa, in agreement with the results presented in previous studies (Sadok and Tamang 2019). Consequently, we investigated the variability of whole-plant TR at a VPD of 2.7 kPa, which is a VPD value higher than the breakpoints of genotypes showing a segmented TR response. This analysis showed that the panel of MAGIC exhibits circa a 5-fold variation of TR as measured values range from 4.05 (genotype “M382”) to 13.6 (genotype “M150”) ${mg}_{H2O}m^{-2}s^{-1}$ (Fig. 1c; Supplementary Table 2).

### Correlation of belowground and physiological traits with GY

**Fig. 2.**
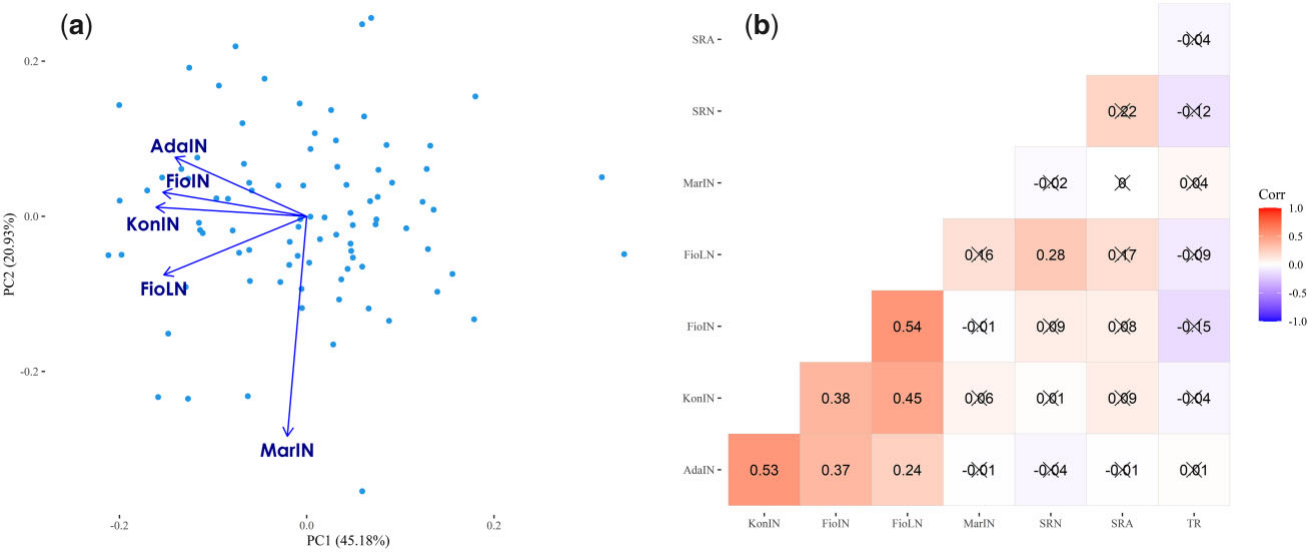
GGE biplot of GY and pairwise correlations of SRA, SRN, GY, and TR at a VPD of 2.7 kPa. a) The environment-vector view of the GGE biplot indicates similarities among test environments in discriminating the genotypes. b) Depending on the trait distribution type (discrete or continuous), values indicate pairwise Pearson’s correlation or polyserial correlation between GY, SRA, SRN, and TR measured under high evaporative demand of VPD (2.7 kPa). Correlation values showing *P*-values larger than 0.05 are marked by a cross.

A genotype × environment (GGE) biplot analysis was carried out using the adjusted means of GY to assess the level of correlation among site-by-season combinations and identify environments with peculiar bio-climatic parameters (Fig. 2a). This analysis indicated that AdaIN and FioIN show the highest environmental similarities, while compared to the remaining environments, MarIN is the most dissimilar one as already substantiated in other studies (Puglisi *et al.* 2021) owing to the dryer and hotter conditions of this site. For assessing whether belowground and physiological traits might contribute to determining yield formation under limiting nitrogen (FioLN) and water conditions (MarIN), we correlated GY with SRN, SRA, and TR measured at a VPD of 2.7 kPa, computing Pearson’s correlation coefficient for pairs of continuous traits (GY, SRA, and TR) and polyserial correlation coefficient for pairs of continuous and discrete (SRN) traits (Fig. 2b). This pairwise correlation analysis indicated that SRN shows a positive and moderate correlation with SRA (*r* = 0.22, *P*-value = 0.07) (Fig. 2b; Supplementary Fig. 1), while GY in FioLN exhibited positive correlations with SRN, showing a value of 0.28 (*P*-value = 0.02) (Fig. 2b; Supplementary Fig. 1). Unexpectedly, no significant correlations were observed between GY measured in MarIN with belowground and physiological traits. Overall, GY showed positive correlations among KonIN, AdaIN, and FioIN, while no significant correlations were observed between MarIN and the remaining sites (KonIN, AdaIN, FioIN, and FioLN) (Fig. 2b).

### Using SRN as proxy trait to predict GY under limiting nitrogen conditions

**Fig. 3.**
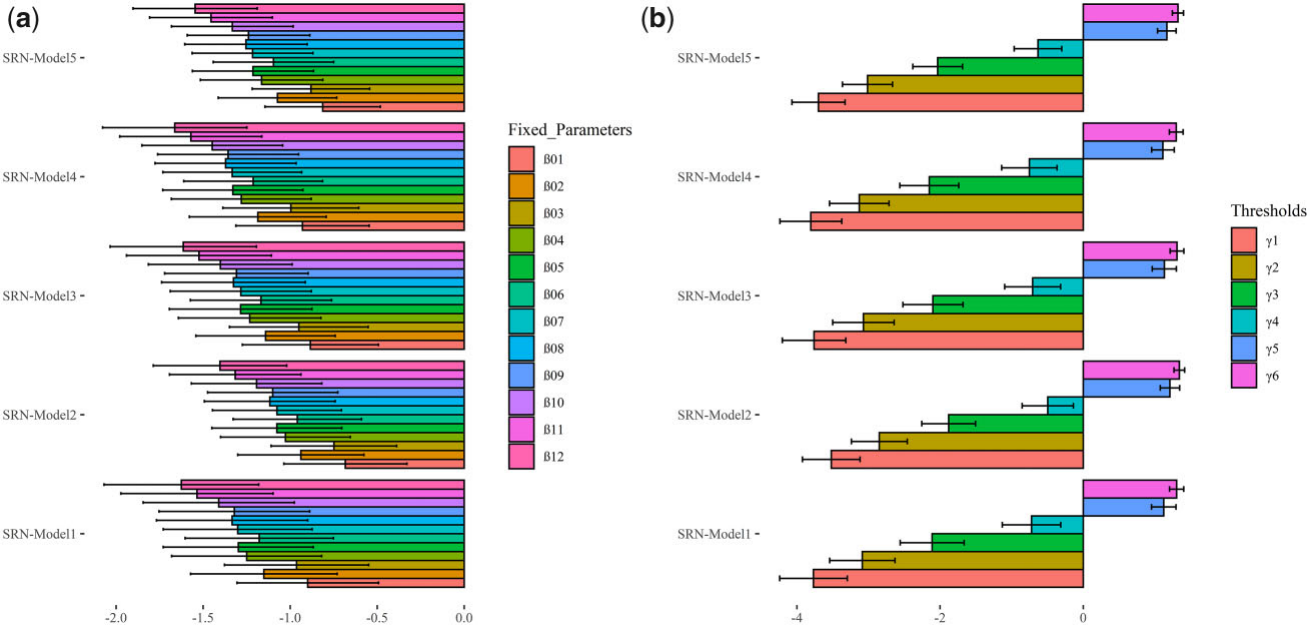
Bar plots of the estimated parameters and thresholds in SRN-Models 1–5. a) Posterior mean and 95% confidence interval of the 12 fixed effects (*β_1_, β_2_,… β_12_*) estimated in the 5 TGBLUP models. b) Posterior mean and 95% confidence interval of threshold parameters ($\gamma_{1},\;\gamma_{2},.\gamma_{6}$). In both graphs, the error bars point out the posterior 95% confidence interval of parameter values.

Here, following the moderate correlation observed between GY obtained under limiting nitrogen conditions and the number of seminal roots in FioLN (Fig. 2b), we assumed that SRN might serve as a proxy trait for predicting GY of MAGIC lines under nitrogen or nutrient deficiency and consequently the number of seminal roots was analyzed as an ordinal categorical phenotype, that is we supposed that genotypes exhibiting less seminal roots are more sensitive to nitrogen deficiency and vice versa. Genotyping data of MAGIC (Puglisi *et al.* 2021) were combined with SRN counted in seedlings for fitting 5 TGBLUP GP models fitted with different sets of linear predictors, which include the fixed effect of replicates (all models), the effect of lines (SRN-Model 1), the effect of molecular markers (SRN-Model 2), the effect of molecular markers and epistasis (SRN-Model 3), the effect of lines and molecular markers (SRN-Model 4), and the effect of lines, markers, and epistasis (SRN-Model 5) [Table 1; Equations (2–6)] (Montesinos-Lopes *et al.* 2015b). SRN-Model 2 represents a standard TGBLUP, while SRN-Model 3, SRN-Model 4, and SRN-Model 5 extend TGBLUP models for including line effects and first-order additive × additive epistasis [Table 1; Equations (2–6)]. The estimates of fixed effects and their 95% confidence intervals, that is the effects of the 12 replicates implemented for phenotyping SRN, showed similar values in the 5 threshold models (Fig. 3a; Supplementary Table 3). Similarly, the estimates of the 6 model thresholds ($\gamma_{1},\;\gamma_{2},\ldots \gamma_{6})$ indicated similar values across the 5 threshold models (Fig. 3b; Supplementary Table 4). Overall, the fitting of SRN-Models 1–5 showed similar posterior means of fixed effects and thresholds (Fig. 3; Supplementary Tables 3 and 4).

**Fig. 4.**
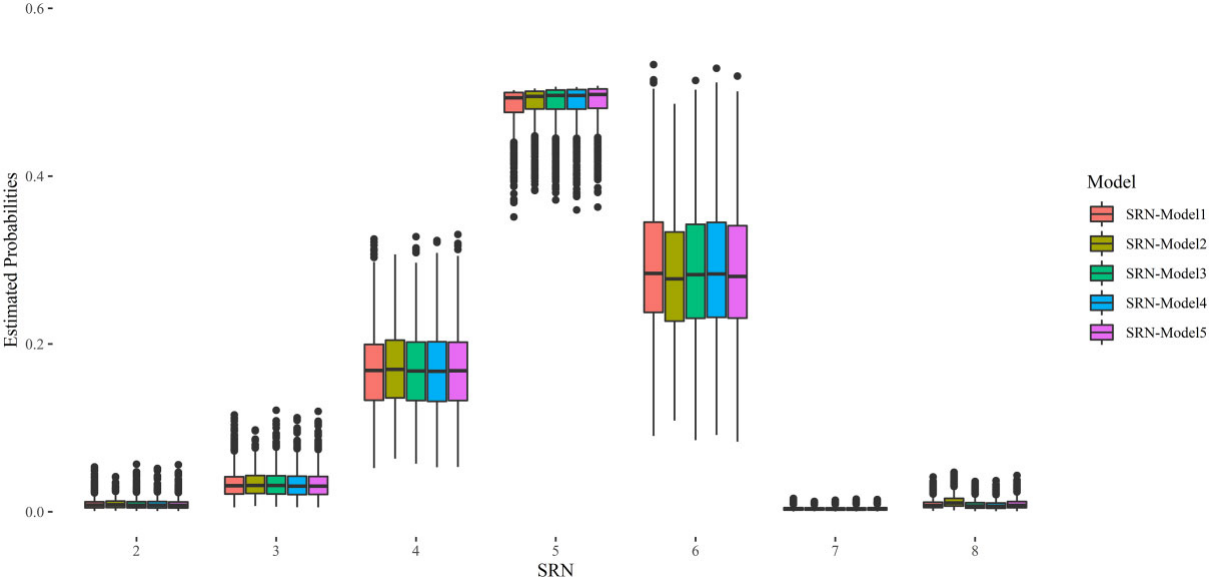
Boxplots of the estimated probabilities of SRN in SRN-Models 1–5. Each boxplot summarizes the distribution of the estimated probability in each category in the 5 TGBLUP models.

The probabilities for each ordinal categorical phenotype estimated in the 5 TGBLUP models for the whole data set are shown in Fig. 4. These boxplots showed that the average probabilities for category 5 (5 seminal roots) are circa 0.50 in the whole data set for all 5 models followed by categories 6 and 4 (Fig. 4). Unlike the distribution estimated from raw data, these probability estimates consider the effect of replicates but, overall, show similar trends from the distributions obtained based on raw frequencies (Fig. 1a).

The analysis of the estimated variance components in the 5 TGBLUP models fitted to SRN data shows that overall, the total variance explained in SRN-Models 1–5 varies from 1.05 to 1.07 (Table 2). Molecular markers explain circa 4.76% of the total variance in SRN-Model 2, and circa 1.8% in SRN-Model 3, 4, and 5 (Table 2). Similarly, the first-order additive × additive epistasis explains 4.76% of the total variance for SRN-Model 3 and 1.88% for SRN-Model 5.

**Table 2.** Estimated variance components of SRN-Models 1–5.

Model	L	G	G × G	Error variance	Total variance
SRN-Model 1	0.07 (6.54%)			1	1.07
SRN-Model 2		0.05 (4.76%)		1	1.05
SRN-Model 3		0.02 (1.86%)	0.04 (4.76%)	1	1.07
SRN-Model 4	0.04 (3.77%)	0.02 (1.88%)		1	1.06
SRN-Model 5	0.02 (1.88%)	0.02 (1.88%)	0.02 (1.88%)	1	1.06

L, estimated variance of line effects; G, estimated variance of marker effects; G × G points out the variance of first-order additive × additive epistasis. Numbers between brackets point out percentage of explained variance of each model predictor.

**Fig. 5.**
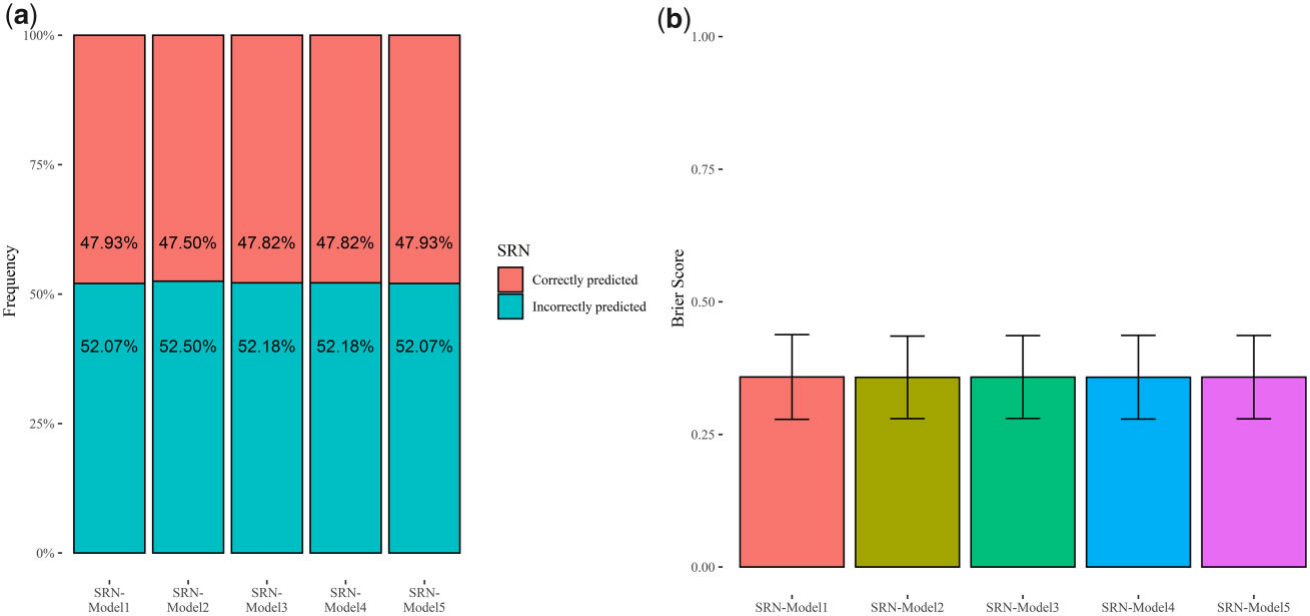
Predictive ability of SRN-Models 1–5. a) Proportion of cases correctly classified using the LOO cross-validation of the 5 TGBLUP models. b) Brier scores obtained from LOO cross-validation of the 5 TGBLUP models.

To assess the predictive ability of SRN-Models 1–5, LOO cross-validation was implemented to compute BS between predicted and observed categorical values and the PCCC (Gianola and Schon 2016; Montesinos-López *et al.* 2020). Cross-validation analysis pointed out the PCCC is circa 50% for the 5 models considered in the present study (Fig. 5a; Supplementary Table 5). Similarly, BS points out a high predictive ability as a value of circa 0.3 was estimated for SRN-Models 1–5 (Fig. 5b).

### GP of SRN using log-normal transformation of count data

**Fig. 6.**
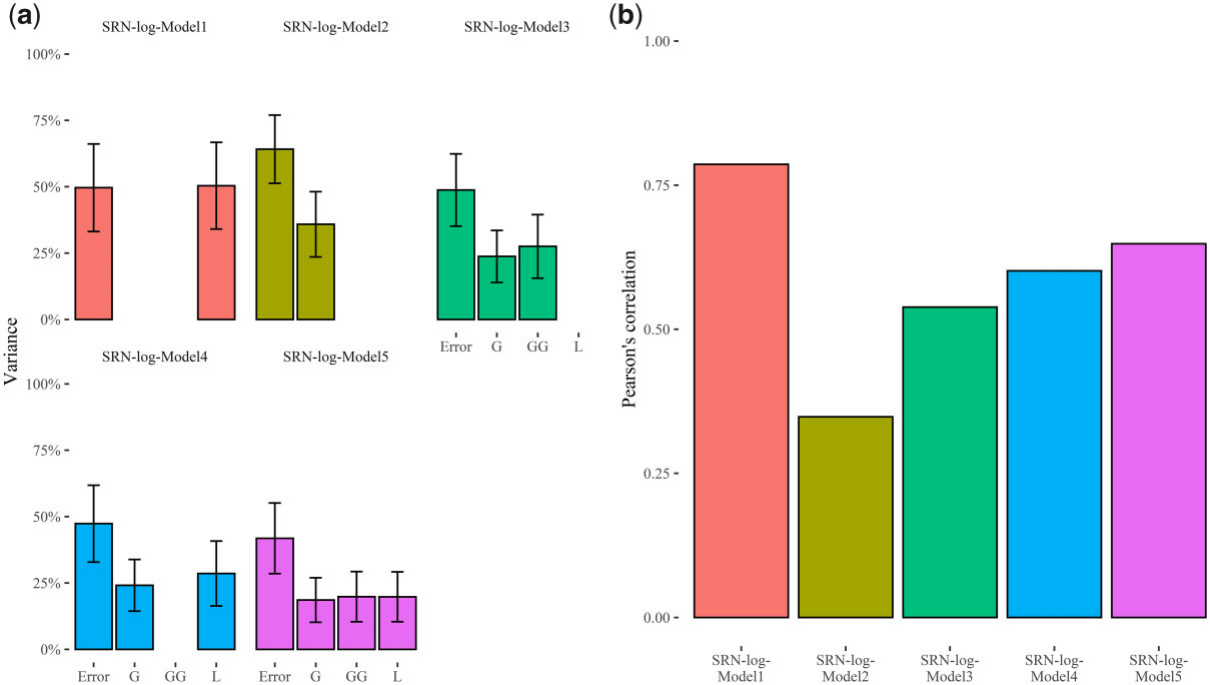
Estimated variance components and predictive ability of SRN-log-Models 1–5. a) Bar plots indicate the explained variance of each component, expressed as percentage of the total model variance. L indicates the estimated variance of line effects; G is the estimated variance of marker effects while G × G and “Error” point out the variance of first-order additive × additive epistatic effects and the residual variance, respectively. Error bars point out the 95% confidence interval of the estimated variances. b) Bar plots of predictive ability values computed as Pearson’s correlation between estimated and observed data using LOO cross validation strategy.

Beyond using SRN as a proxy trait for predicting GY under nutrient scarcity, GP models for count data were fitted to predict this trait independently from its association with drought tolerance and GY under nitrogen deficiency at the mature stage, using log-normal models (SRN-log-Models 1–5) incorporating the same combinations of linear predictors included in SRN-Models 1–5 (Table 1). The analysis of variance components of these 5 log-normal models showed that “SRN-log-Model 5,” which incorporates line (L), marker (G), and first-order additive × additive epistasis (G × G), has a lower error variance compared to the other models considered in the present study, and allows to better fit the data (Fig. 6a; Supplementary Table 6). The variance of G × G is 27.52% for SRN-log-Model 3 and 19.83% for SRN-log-Model 5 (Fig. 6a; Supplementary Table 6). LOO cross validation pointed out that the predictive ability values of these models, measured using Pearson’s correlation coefficient between predicted and observed data, range from 0.35 (SRN-log-Model 2) to 0.79 (SRN-log-Model 1), while SRN-log-Model 3, SRN-log-Model 4, and SRN-log-Model 5 show predictive ability values of 0.54, 0.60, and 0.65, respectively (Fig. 6b; Supplementary Table 6). Overall, model comparison indicated that for SRN, log normal models that explicitly incorporate markers and first-order additive × additive epistatic interactions capture a larger fraction of the total phenotypic variability and have better predictive ability.

### Prediction of SRA in barley seedlings

**Fig. 7.**
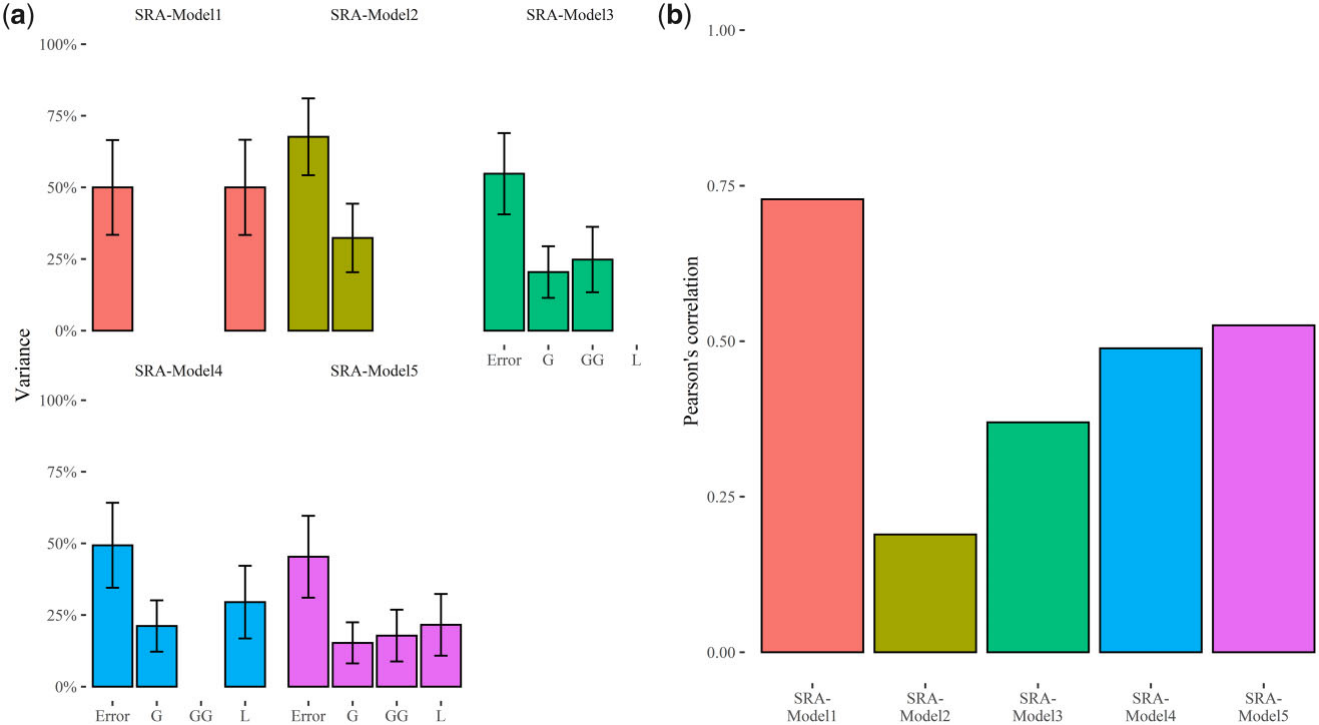
Estimated variance components and predictive ability of the SRA-Models 1–5. a) Bar plots indicate the variance of each component, expressed as percentage of the total model variance. L indicates the estimated variance of line effects; G is the estimated variance of marker effects while G × G and “Error” point out the variance of additive × additive epistatic effects and the residual variance, respectively. Error bars point out the 95% confidence interval of the estimated variances; b) bar plot of predictive ability values computed using Pearson’s correlation between estimated and observed SRA.

As with log-normal models, the GBLUP counterpart of the 5 TGBLUP models used for predicting SRN were fitted to SRA. These 5 models incorporate the main effects and interactions used for SRN [Table 1; Equations (7–11)] but assume that the response variable, that is SRA, is continuous and follows a normal distribution. The adjusted means of SRA were combined with 20,426 polymorphic SNPs detected in this panel of MAGIC population to fit these extended GBLUP models. The analysis of variance components of these 5 models showed that “SRA-Model 5,” which incorporates line, marker, and first-order additive × additive epistatic interaction effects, has a lower error variance compared to the other models considered in the present study and allows to better fit data (Fig. 7a; Supplementary Table 7). The variance of first-order additive × additive epistatic interaction was 27.79% for SRA-Model 3 and 17.81% for SRA-Model 5 (Fig. 7a; Supplementary Table 7). LOO cross validation pointed out that the predictive ability values of these models, measured using Pearson’s correlation coefficient between predicted and observed data, range from 0.19 (SRA-Model 2) to 0.73 (SRA-Model 1), while SRA-Model 3, SRA-Model 4, and SRA-Model 5 show predictive ability values of 0.37, 0.49, and 0.53, respectively (Fig. 7b; Supplementary Table 7). As observed for log-normal models (SRN-log-Models 1–5), models that predict SRA incorporating marker and first-order epistatic effects in the linear predictors show higher predictive ability and better model fitting.

### Models for predicting TR under high evapotranspiration demand

**Fig. 8.**
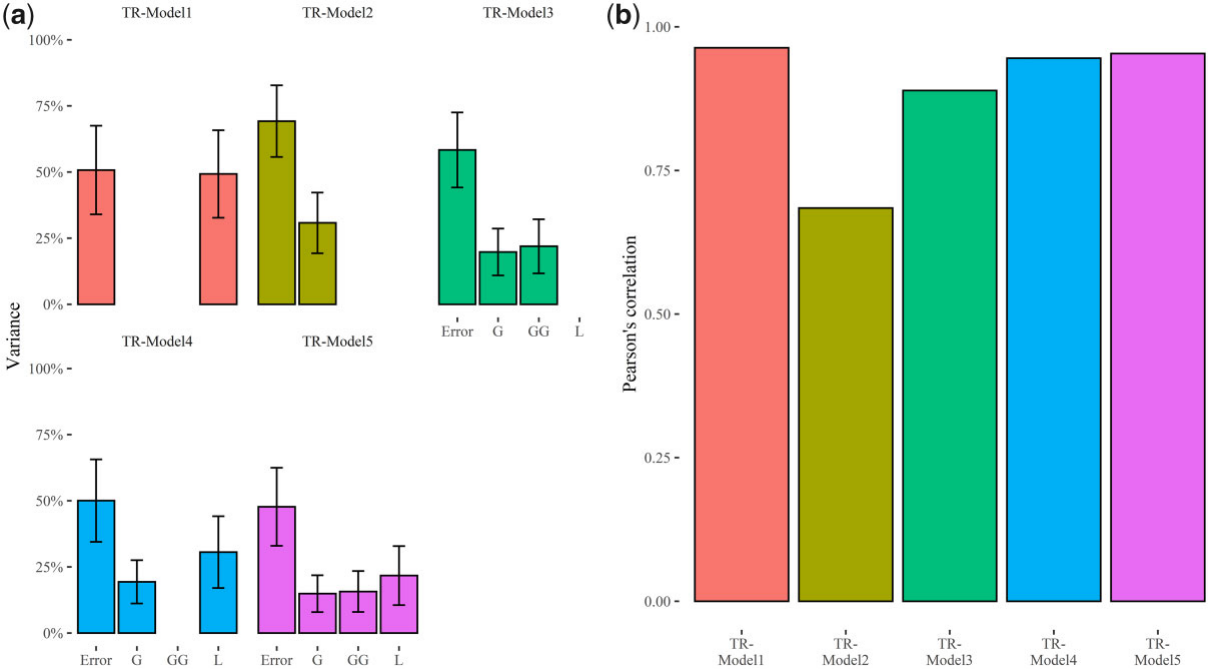
Estimated variance components and predictive ability of the TR-Models 1–5: a) bar plots indicate the variance of each component, expressed as percentage of the total model variance. L indicates the estimated variance of line effects; G is the estimated variance of marker effects, while G × G and “Error” point out the variance of additive × additive epistatic effects and the residual variance, respectively. Error bars point out the 95% confidence interval of the estimated variances; b) bar plot of predictive ability values expressed as Pearson’s correlation between estimated and observed TR under high evaporative demand (2.7 kPa).

The 5 combinations of linear predictors incorporated in GP models fitted to SRA and SRN data [Table 1; Equations (12–16)] were used to predict TR at a VPD of 2.7 kPa. The analysis of variance components of these 5 models showed that “TR-Model 5,” which incorporates line, marker, and first-order additive × additive epistatic interaction effects as linear predictors, has a lower error variance compared to the other models considered in the present study and allows to better fit the data (Fig. 8a; Supplementary Table 8). The analysis of variance components showed that first-order additive × additive epistatic interaction effects explain 21.93% and 15.69% of the total variance for TR-Model 3 and TR-Model 5, respectively (Fig. 8a; Supplementary Table 8). LOO cross validation pointed out that the predictive ability values of these models, measured using Pearson’s correlation coefficient between predicted and observed data, range from 0.68 (TR-Model 2) to 0.96 (TR-Model 1), while TR-Model 3, TR-Model 4, and TR-Model 5 show predictive ability values of 0.89, 0.95, and 0.95, respectively (Fig. 8b; Supplementary Table 8). Like observed for GP models fitted to belowground traits, this analysis demonstrated that models that explicitly incorporate marker and interaction effects fit better the TR data and have better predictive ability.

## Discussion

In the present study, the phenotypic variability of SRN, SRA, and TR under increasing evaporative demand was surveyed in a panel of MAGIC lines of barley (Supplementary Tables 1 and 2). The results showed that, in this genetic material, SRN can vary from 2 to 8 (Fig. 1a), consistently with other studies carried out on populations of mostly unrelated accessions of barley (Robinson *et al.* 2016; Jia et al. 2019).

In the scientific literature, protocols for SRA phenotyping propose to examine the first pair of seminal roots (Robinson *et al.* 2016) or the 2 outmost seminal roots (Jia et al. 2019). Following the first protocol, in the present study, SRA between the first pair of seminal roots has been measured, and the comparison of our results with those obtained in other works carried out following the same methodology (Robinson *et al.* 2016, 2018) points out that the SRA has a wider phenotypic distribution in MAGIC lines. On the other side, the lack of common genotypes between these experiments hampers our ability to compare results across different studies and does not allow to attribute the wider distribution of SRA detected in our genetic material to confounding or genetic effects. Unlike the findings reported in other studies (Robinson *et al.* 2016), SRA and SRN measured in barley seedlings showed a moderate correlation (*r* = 0.22; Fig. 2b), slightly above the threshold of significance (*P*-value = 0.07; Supplementary Fig. 1). Nevertheless, this comparison is not exhaustive as in other studies SRN was handled as a continuous trait and the phenotypic variability was presented using the adjusted means (BLUE or BLUP), while in this research work SRN was analyzed as a discrete trait. The analysis of SRN as continuous or discrete phenotypic trait implies different assumptions, which in turn hamper the comparisons of trait correlations; as in our analysis, SRN was considered as a discrete trait and consequently the polyserial correlation between SRA and SRN was computed instead of using Pearson’s correlation coefficient. Interestingly, the present study indicated a moderate and positive and significant correlation of SRN (*r* = 0.28; *P*-value = 0.02) with GY measured in FioLN, which was managed with reduced amount of nitrogen (Fig. 2b; Supplementary Fig. 1). As highlighted for other cereal crops, this correlation suggests a link between the root architecture system and the ability of barley to grow in soils with a reduced nitrogen fertilization without experiencing yield penalty. Anyway, the sample size of this analysis along with the inclusion of only 2 site-by-season combinations organized with this nitrogen management impose to carry out other studies to definitively underpin the tie between these belowground traits measured at the seedling stage and the ability to promote yield formation under limiting nitrogen conditions.

In recent years, TR has been widely targeted in different crops to exploit its correlation to drought tolerance (Schoppach and Sadok 2012, 2013; Schoppach *et al.* 2016). Nevertheless, in barley, the analysis of TR in response to high evaporative demand has lagged behind and to date has been investigated on a limited panel of 25 wild barley and in 1 cultivar (Sadok and Tamang 2019), which showed that at a VPD of circa 2.7 kPa, TR ranges from circa 25 to 75 ${mg}_{H2O}m^{-2}s^{-1}$ depending on the genotype. Our study confirms that in barley, the TR at high evaporative demands is significantly lower than the values observed in other cereal crops (Schoppach and Sadok 2012; Sinclair *et al.* 2017; Tamang and Sadok 2018) and that the TR measured in our MAGIC population exhibits lower values of TR compared to other results obtained in barley at the same VPD values (Sadok and Tamang 2019). As substantiated with SRA and SRN analyses, technical causes and the lack of common genotypes hamper cross-study comparison of TR in different barley germplasm, but it is plausible that, in general, our MAGIC population has a lower TR response to high evaporative demand compared to the barley genotypes investigated in other studies (Sadok and Tamang 2019).

Unexpectedly, our analyses did not detect significant correlation between TR and GY measured in MarIN, which is the hottest and driest environment investigated in this study pointing out that, in barley, this trait might exhibit correlation with GY under harsher conditions. In field trials, GY depends on the genotypic values of plants and environmental factors that exert their influence from sowing to harvest and consequently it is not surprising to detect inconsistent correlations with the belowground and physiological traits examined in the present study. The present study does not address the correlations between SRN, SRA, and TR measured at the juvenile stage and the root architecture of mature barley, which remain still unclear and deserve additional analyses.

In the present study, genomic-enabled prediction of SRA, SRN, and TR measured at a VPD of 2.7 kPa was carried out, leveraging on prediction models for different types of data (continuous, count, and ordinal) and we showed that these traits can be modeled with high predictive ability. These findings are relevant for barley improvement and ideotype breeding as can pave the way to exploit untapped plant collection minimizing phenotyping costs. For standard, log normal, and TGBLUP GP models, we used 5 sets of linear predictors, which differ for the types of effects considered (Table 1). Using TGBLUP models, we observed that the inclusion of additive × additive epistatic interaction (G × G) in the set of linear predictors (SRN-Model 3 and SRN-Model 5) increases the total variance that models account for (Table 2). These results are corroborated in log-normal GP models, as also these models benefit of the inclusion of additive × additive epistatic interaction in the set of linear predictors (Figs. 6–8). Overall, for all traits examined in the present study, we found that the inclusion of interaction effects brings advantages both in model fitting and predictive ability, as already substantiated in several studies (Varona *et al.* 2018). Despite being called additive × additive epistatic interactions, the functional interpretation of these effects and of the variance component counterpart may be misleading as their existence in nonadditive GP models and genome-wide association studies does not prove the role of epistasis in the actual genetic architecture of these traits (de los Campos *et al.* 2019; Schrauf *et al.* 2020). Particularly, the low marker density used in the present study to fingerprint the panel of MAGIC and the incomplete linkage disequilibrium of markers (Puglisi *et al.* 2021) favor the detection of phantom epistasis in nonadditive GP models, that is the portion of additive effects that is not captured in the models due to incomplete linkage disequilibrium generates apparent epistasis, which is in turn detected in our models including additive × additive epistatic interactions (de los Campos *et al.* 2019; Schrauf *et al.* 2020). While the functional interpretation of first-order additive × additive effects might be questionable, the inclusion of epistatic effects in GP should be considered in conditions that foster the emergence of apparent epistasis to improve predictive ability of models (Schrauf *et al.* 2020).

Several traits that are relevant for plant breeding are not normally distributed and need to be analyzed using special statistical techniques (Montesinos-López *et al.* 2015a). Traits that fall in this category are proportion of plants that overcome a stress, disease resistance scored using discrete scales, and SRN. In the present study, we used TGBLUP models assuming that SNR varies as an ordinal discrete variable indicating the ability of plants to grow under water or nutrient scarcity. (Montesinos-López *et al.* 2015a). Despite the low values of variance explained by molecular markers (Table 2), the TGBLUP models fitted to SRN showed high predictive ability values (Brier Score equals to 0.36) (Fig. 5), highlighting that GP can be successfully applied to traits showing low heritability as already substantiated in other studies carried out in plants (Zhang *et al.* 2017; Klápště *et al.* 2020) and animals (Guo *et al.* 2014; Iheshiulor *et al.* 2016).

Count data arise in plant breeding when the trait of interest is the sum of discrete quantities that can take only integer values (e.g. the number of tillers per plant or the number of seminal roots in seedlings). Currently, for this type of data, GP models are fitted using standard GBLUP along with log transformed data or specific generalized mixed linear models that use a link function based on Poisson or Negative Binomial distributions (Montesinos-López *et al.* 2016). This latter group of models have been recently formalized, implemented in a Bayesian framework, and compared with log-normal models for count data (Montesinos-López *et al.* 2016). In the present study, GP for SRN was modeled as count data fitting log-normal GP models as preliminary tests showed that the computational demand of these models does not counterbalance the higher predictive ability of these models. Multitrait GP might be a natural approach to exploit SRA, SRN, and TR response at high VPD as multivariate GP has been shown, using either real or simulated data sets, to have superior predictive ability compared to univariate GP. While the use of traits varying on a continuous scale is straightforward in multivariate GP, count data still pose several challenges that currently hamper our ability to exploit this trait in multivariate analyses.

## Data availability

The phenotypic data underlying this article are available in the online Supplementary material (Supplementary Tables 1 and 2 File 2). The genotyping data underlying this article are available in the comma delimited file named “Supplementary File 1,” which contains the genotypes detected at 20,426 loci.

Supplemental material available at *G3* online.

## Supplementary Material

jkac022_Supplemental_Text

jkac022_File_S1

jkac022_File_S2

jkac022_Figure_S1
