# Supplementary material for: High accuracy of genome-enabled prediction of belowground and physiological traits in barley seedlings
Source: G3 (Bethesda). 2022 Jan 31;12(3):jkac022. doi: 10.1093/g3journal/jkac022 (PMC8895982; doi:10.1093/g3journal/jkac022)
Supplement: jkac022_Supplemental_Text [file jkac022_supplemental_text.docx]

# Supplementary text

## S1 Procedure for deriving the adjusted means of grain yield

All field trials were analyzed using an alpha-lattice experimental design with two replicates and adjusted means of GY were computed using a mixed linear model considering genotypes as random effects (Puglisi et al. 2021). Mixed linear models for analyzing field trial data were implemented in lme4 package (Bates et al. 2015) along with R 4.0.3 (Core R Team 2019) and used to compute the best linear unbiased predictions (BLUPs) of genotypic effects per environment and per site-by-season-by-management combination including both HD and year as covariates, respectively. The adjusted means of GY were centered and standardized for all subsequent analyses (**File S2**). The adjusted means of GY were used to study genotype × environment (GE) interaction for delineating clusters of mega-environments using genotype plus genotype × environment (GGE) biplot analyses (Yan et al. 2000; Yan and Holland 2010) implemented in GGEBiplots package and for assessing correlation with physiological and belowground traits.

## S2 Procedure for phenotyping SRA and SRN and deriving the adjusted means of SRA

To phenotype SRA and SRN, the set of 90 MAGIC genotypes was randomized in 45 pots using 12 replicates per genotypes. In each pot 24 random genotypes were sowed at a distance of 2.5 $cm$ from each other and at a depth of circa 1.5-2 $cm$, positioning the embryo towards the bottom of the pot to support the correct root development. After sowing, pots were irrigated and placed inside black container pots, in a lightless growing chamber with controlled climate conditions including two data loggers, at 19 °C and 70% of relative humidity (Type TGU-4550, Gemini Data Loggers, UK). Five days after sowing twelve images of SRA per genotype were acquired with a digital camera, which were subsequently analyzed using ImageJ software (<https://imagej.nih.gov>) (Schneider et al. 2012) to measure the angle between the first pairs of seminal roots according to published protocols (Richard et al. 2015). Eleven-day days after sowing seedlings were carefully removed from soil and SRNs were manually counted in the same order used for measuring SRA. The adjusted means of SRA and their 95% confidence interval were estimated in R 4.0.3 statistical (Core R Team 2019) using lme4 package (Bates et al. 2015) analyzing the experimental design used for phenotyping SRA with the following linear model:

$y_{ijk} = 1\mu+{Rep}_{i} +{Pot}_{j} +{Gen}_{k}+e_{ijk}$ **Equation 1**

where $y_{ijk}$ is the response variable, that is the raw SRA data, µ is the general mean, ${Rep}_{i}$ is the fixed effect of the $i^{th}$ replicate, ${Pot}_{j}$ is the random effect of the$j^{th}$ pot which was assumed to be distributed multivariate normally with mean 0 and unstructured covariance matrix, that is ${Pot}_{i}\sim N(0,\Sigma$), ${Gen}_{k}$ is the fixed effect of the $k^{th}$ genotype and $e_{ijk}$ is the error associated to each response, which was supposed to independent and identically distributed, that is $e_{ijk}\sim N(0,\sigma_{e}^{2})$. The adjusted means of SRA computed with this model (Equation 1) were used to fit censored GP models and for seeking correlations with other belowground and physiological traits.

## S3 Procedure for estimating the TR at different values of VPD

To determine the amount of water loss by transpiration, the weight of pots was measured using an electronic balance with a resolution of 0.1 $g$ (KB Kern 573, Kern & Sohn GmbH, Balingen, Germany), at seven values of VPD. For calculating evapotranspiration, the weight of five empty pots was measured seven times in parallel with the experiment and at the day after plants leaf areas were immediately collected using leaf area meters (LI-3000C, LiCor Biosciences, Lincoln, Nebraska, USA) and data loggers (Type TGU-4550, Gemini Data Loggers, UK). The VPD applied to plants was computed as a function of temperature and humidity and TR measures in ${mg}_{H2O}m^{-2}s^{-1}$ was normalized as function of total leaf area and time in which pot weight was collected.

TR data were regressed on VPD values using different models. To determine the trend of TR as function of VPD, linear (**Equation 2**) and segmented models (**Equations 2** **and 3**) were fitted to the phenotyping data (Medina et al. 2019) as follows:

$y_{1}= S_{1}x + I_{1}$ **Equation 2**

$y_{2}= S_{2}x + I_{2}$ **Equation 3**

where$S$ and $I$ point out slope and intercept, respectively. These models were computed using “segmented” package (V. R. M. Muggeo 2008; Muggeo 2017) implemented in R 4.0.3 statistical (Core R Team 2019). Goodness-of-fit based on R-squared values was used as main criterion to select the best model and classify genotypes in a first group of lines showing linear trend of TR and a second group showing a segmented trend.

Subsequently, the raw data of TR measured at 2.7 $KPa$ of VPD were analyzed using the following linear model to compute the corresponding adjusted means:

$y_{ik} = 1\mu+{Rep}_{i}+{Gen}_{k}+e_{ik}$ **Equation 4**

where $y_{ik}$ are the raw TR values, *µ* is the general mean, ${Gen}_{k}$ is the fixed effect of the $k^{th}$ genotype and $e_{ik}$ is the error associated to each response, which was supposed to independent and identically distributed, that is $e_{ijk}\sim N(0,\sigma_{e}^{2})$. The random effects ${Rep}_{i}$ are assumed to be distributed multivariate normally with mean 0 and unstructured covariance matrix, that is ${Rep}_{i}\sim N(0,\Sigma$). The adjusted means of TR measured at low levels of VPD along with their 95% confidence intervals were estimated in R 4.0.3 statistical (Core R Team 2019) using lme4 package (Bates et al. 2015) and used to fit censored GP models and compute broad sense heritability (H^2^).

## References

Bates D, Mächler M, Bolker BM, Walker SC (2015) Fitting linear mixed-effects models using lme4. J Stat Softw 67:. https://doi.org/10.18637/jss.v067.i01

Core R Team (2019) A Language and Environment for Statistical Computing. R Found. Stat. Comput. 2:https://www.R--project.org

Medina S, Vicente R, Nieto-Taladriz MT, et al (2019) The plant-transpiration response to vapor pressure deficit (VPD) in durum wheat is associated with differential yield performance and specific expression of genes involved in primary metabolism and water transport. Front Plant Sci 9:1–19. https://doi.org/10.3389/fpls.2018.01994

Muggeo VMR (2017) Interval estimation for the breakpoint in segmented regression: a smoothed score-based approach. Aust New Zeal J Stat 59:311–322. https://doi.org/10.1111/anzs.12200

Richard CAI, Hickey LT, Fletcher S, et al (2015) High-throughput phenotyping of seminal root traits in wheat. Plant Methods 11:1–11. https://doi.org/10.1186/s13007-015-0055-9

Schneider CA, Rasband WS, Eliceiri KW (2012) NIH Image to ImageJ: 25 years of image analysis. Nat Methods 9:671–675. https://doi.org/10.1038/nmeth.2089

V. R. M. Muggeo (2008) segmented: An R Package to Fit Regression Models with Broken-Line Relationships. R News 3:343–4

Yan W, Holland JB (2010) A heritability-adjusted GGE biplot for test environment evaluation. Euphytica 171:355–369. https://doi.org/10.1007/s10681-009-0030-5

Yan W, Hunt LA, Sheng Q, Szlavnics Z (2000) Cultivar evaluation and mega-environment investigation based on the GGE biplot. Crop Sci 40:597–605. https://doi.org/10.2135/cropsci2000.403597x

**Supplementary Figures**

**Figure S1: *P* values related to the pairwise correlations of SRA, SRN, GY and TR at a VPD of 2.7 KPa.** *P* values less than 0.05 are reported in blue.


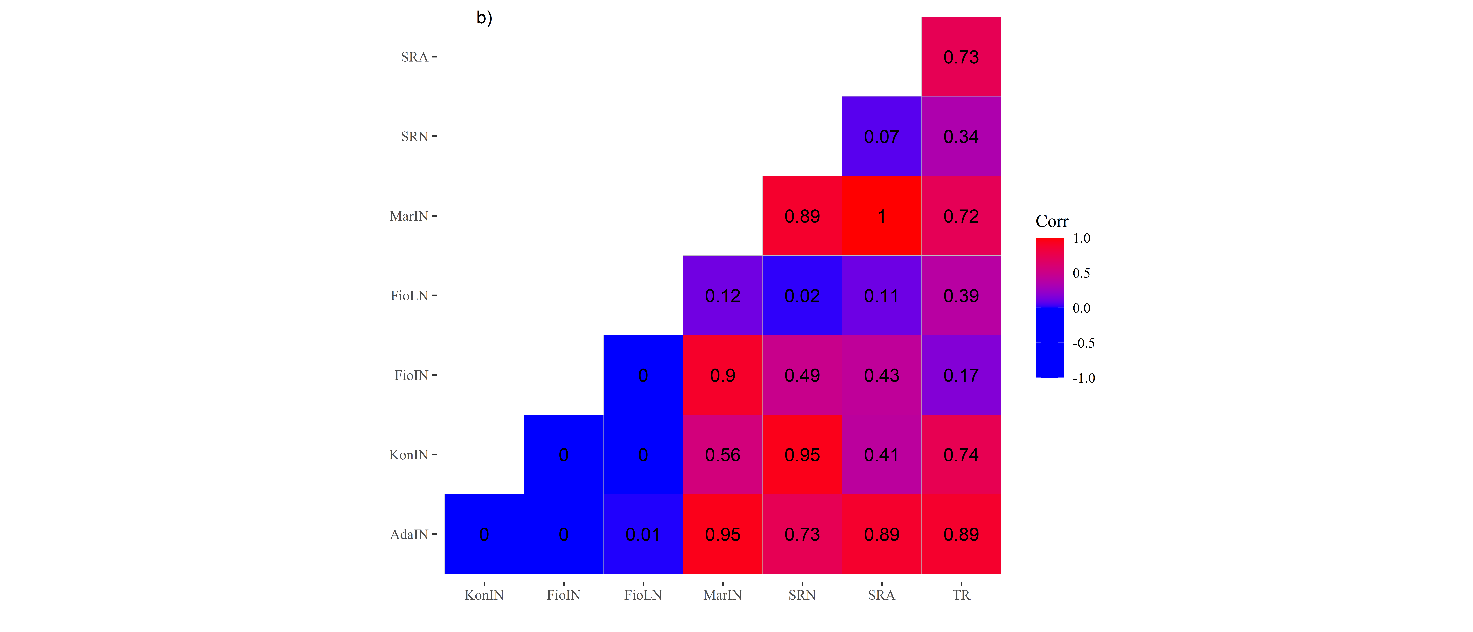


**Supplementary Tables**

**Table S1: Phenotypic data obtained for the panel of MAGIC lines.** For each line, the raw data of SRN was reported. Maximum (Max), minimum (Min) average (Mean) values were calculated at the end of the table.

| Genotype | Rep 1 | Rep 2 | Rep 3 | Rep 4 | Rep 5 | | Rep 6 | Rep 7 | Rep 8 | Rep 9 | Rep 10 | Rep 11 | Rep 12 |
| --- | --- | --- | --- | --- | --- | --- | --- | --- | --- | --- | --- | --- | --- |
| Aldebaran | 5 | 5 | 5 | 5 | 4 | | 5 | 3 | 4 | 4 | 5 | 3 | 5 |
| Athene | 6 | 5 | 6 | 4 | NA | | 4 | 6 | 4 | NA | 5 | 5 | 5 |
| Dea | 5 | 5 | 5 | 6 | 5 | | 5 | 4 | 5 | 5 | 6 | 5 | 5 |
| Fridericus | 4 | 4 | 5 | 5 | 5 | | 5 | 4 | 5 | 5 | 4 | 2 | 4 |
| Hatif de Grignon | 5 | 5 | 6 | NA | 5 | | 5 | NA | 6 | NA | 6 | 5 | NA |
| Ketos | 5 | 5 | 6 | 5 | 5 | | 4 | 6 | 5 | 3 | 3 | 4 | 5 |
| Ponente | 6 | 5 | 5 | 5 | 6 | | 6 | 6 | 4 | 4 | 5 | 5 | 4 |
| Robur | 5 | 6 | 6 | 6 | 6 | | NA | 6 | 3 | 5 | 6 | 6 | 5 |
| M101 | 5 | 5 | 4 | 5 | 5 | | 5 | 5 | 4 | 6 | NA | 6 | 5 |
| M102 | 4 | 6 | 5 | NA | NA | | 5 | 5 | 5 | 4 | NA | 4 | 5 |
| M106 | 6 | 6 | 4 | 5 | 5 | | 5 | 4 | 5 | 2 | 6 | 5 | 3 |
| M11 | 5 | 4 | 5 | 4 | 4 | | 5 | NA | NA | NA | 6 | 4 | NA |
| M118 | 5 | 6 | 6 | 6 | 6 | | 4 | 6 | 5 | 5 | NA | 6 | 6 |
| M121 | 6 | 6 | 6 | 5 | 5 | | 5 | 6 | 4 | 4 | 5 | 5 | 5 |
| M131 | 5 | 4 | 6 | 6 | NA | | 4 | 6 | 6 | 5 | 4 | 7 | NA |
| M132 | 5 | 4 | 5 | 4 | 5 | | 6 | NA | 5 | 6 | 5 | NA | 5 |
| M136 | 6 | NA | 5 | 5 | 5 | | 5 | 6 | NA | 5 | 4 | NA | NA |
| M140 | 4 | 5 | 6 | 4 | 6 | | 5 | 6 | 6 | 5 | 5 | 5 | 6 |
| M147 | 6 | 6 | 6 | 5 | 4 | | 5 | 3 | 4 | NA | 5 | 5 | 4 |
| M148 | 4 | 6 | 5 | 5 | 5 | | 5 | NA | 6 | 6 | 5 | 6 | 4 |
| M149 | 5 | 5 | 6 | 5 | 5 | | 6 | 6 | NA | 6 | 4 | 5 | 2 |
| M150 | 5 | 5 | 5 | 6 | 6 | | 6 | 6 | NA | 5 | NA | 5 | 6 |
| M151 | 6 | 6 | 5 | 4 | 4 | | 5 | 5 | 5 | 5 | 6 | 4 | 4 |
| M154 | 5 | 5 | 6 | 5 | 5 | | 5 | 5 | 5 | 5 | 5 | 5 | 5 |
| M156 | 6 | 5 | 6 | 5 | 5 | | 5 | 5 | 6 | 5 | 2 | 6 | NA |
| M158 | 5 | 5 | 5 | 5 | 4 | | 5 | 5 | 6 | 5 | 5 | 5 | 4 |
| M162 | 5 | 4 | 6 | 4 | NA | | 4 | 5 | 4 | 6 | 4 | 5 | 4 |
| M163 | 6 | 5 | 6 | 5 | 4 | | 6 | NA | 6 | 6 | 5 | 6 | 5 |
| M169 | NA | 5 | 6 | 6 | 5 | | 4 | 6 | 5 | 6 | 4 | 5 | 5 |
| M177 | 5 | 4 | 5 | 4 | 5 | | 5 | 4 | 4 | 5 | 5 | 4 | 5 |
| M18 | 6 | 5 | 5 | 5 | 4 | | 5 | 5 | NA | 5 | 5 | 5 | NA |
| M180 | 6 | 4 | 5 | 5 | 4 | | 6 | 6 | 4 | 6 | 5 | 4 | 4 |
| M181 | 5 | 6 | 5 | 5 | 3 | | 6 | 3 | 5 | 6 | 5 | 3 | 6 |
| M190 | 5 | 6 | 5 | 5 | NA | | 6 | 5 | 5 | 4 | 5 | 5 | 6 |
| M194 | 6 | 5 | 6 | 6 | 5 | | 6 | 6 | 6 | 5 | 6 | 5 | 3 |
| M200 | 5 | 5 | 4 | 5 | 4 | | 5 | 3 | 4 | 4 | 4 | NA | 4 |
| M201 | 5 | 6 | 6 | 5 | 5 | | 5 | 4 | 5 | 5 | 5 | 5 | 5 |
| M215 | 6 | 5 | 5 | 5 | 6 | | 5 | 5 | 5 | 5 | 6 | 6 | 5 |
| M216 | 6 | 4 | 5 | 5 | 5 | | 6 | 6 | 5 | 4 | 5 | 5 | 6 |
| M22 | 4 | 6 | 4 | 4 | 5 | | 3 | 3 | 5 | 4 | NA | 4 | 4 |
| M224 | 6 | 4 | 5 | 5 | 6 | | 6 | 5 | 5 | 5 | 6 | 5 | 5 |
| M233 | 6 | 5 | NA | 6 | NA | | 6 | 6 | 5 | 6 | 4 | 5 | 6 |
| M237 | 4 | NA | 4 | 5 | 5 | | 6 | 2 | 6 | 5 | 6 | 6 | 4 |
| M242 | 5 | 5 | 4 | 5 | 6 | | 5 | 5 | 5 | 4 | 5 | 5 | 5 |
| M246 | 5 | 6 | 6 | 4 | 5 | | 5 | 6 | 5 | 4 | NA | 4 | NA |
| M253 | 5 | 6 | 6 | 6 | 6 | | 6 | 6 | NA | 5 | 5 | 4 | 6 |
| M254 | 6 | 6 | 5 | 6 | 5 | | 5 | 3 | 3 | 4 | 7 | 3 | 5 |
| M258 | 5 | 5 | 5 | 5 | 5 | | 5 | 5 | 5 | 5 | 5 | 5 | NA |
| M259 | 6 | 5 | 5 | 5 | 6 | | 5 | 3 | 6 | 5 | 6 | 5 | 4 |
| M262 | 6 | 5 | 4 | NA | 6 | | 4 | 6 | 5 | 4 | 4 | 5 | 6 |
| M264 | 5 | 5 | 5 | 5 | NA | | 5 | 6 | 6 | NA | 5 | NA | NA |
| M278 | 5 | 6 | 5 | 5 | 3 | | 6 | 6 | 4 | NA | 5 | 3 | 5 |
| M281 | 6 | 4 | 5 | 5 | 4 | | 6 | 5 | 5 | NA | 6 | 4 | 5 |
| M285 | 6 | 6 | 5 | 6 | 6 | | 6 | 6 | NA | 6 | 4 | 6 | 5 |
| M287 | 5 | 6 | 6 | 6 | 5 | | 5 | 4 | 5 | 6 | 3 | NA | 4 |
| M288 | 6 | 6 | 6 | 5 | NA | | 6 | 6 | 5 | 5 | NA | NA | 5 |
| M289 | 5 | 8 | 5 | 5 | 4 | | 3 | 5 | 5 | 6 | 5 | 5 | 6 |
| M298 | 6 | 6 | 5 | 6 | 5 | | 6 | 5 | 6 | 6 | 6 | 6 | 6 |
| M299 | 6 | 4 | 6 | NA | 4 | | 5 | 5 | 5 | 6 | 5 | 5 | 5 |
| M30 | 5 | 5 | 4 | 6 | 5 | | 5 | 5 | 5 | 5 | 5 | 5 | 4 |
| M304 | 4 | 4 | 5 | 5 | 5 | | 5 | 5 | 5 | 4 | 4 | NA | 5 |
| M313 | 7 | 6 | 5 | 6 | 6 | | 4 | 5 | 5 | 5 | 5 | 5 | 4 |
| M314 | 6 | 6 | 6 | NA | 6 | | 6 | 5 | 6 | 6 | 5 | NA | 5 |
| M321 | 5 | 5 | 5 | 5 | 6 | | 5 | 6 | 6 | 6 | 5 | NA | 4 |
| M324 | 6 | 5 | 5 | 5 | 4 | | 5 | 6 | 5 | 5 | 5 | 5 | 5 |
| M328 | 5 | 6 | 6 | 5 | 6 | | 5 | 6 | 5 | 6 | 5 | 4 | 4 |
| M332 | 6 | 5 | 5 | 6 | NA | | 6 | NA | 6 | 6 | 5 | 5 | 5 |
| M338 | 5 | 6 | 5 | 5 | 5 | | 5 | 5 | 4 | NA | 5 | 5 | 5 |
| M339 | 5 | 5 | 6 | 4 | 6 | | 4 | 5 | 6 | 5 | 5 | 6 | 5 |
| M345 | 5 | NA | 6 | 6 | NA | | 6 | 3 | 6 | 6 | 5 | 5 | 6 |
| M349 | 6 | 4 | 5 | NA | 3 | | 5 | 6 | 4 | NA | 6 | 6 | 4 |
| M350 | 6 | 6 | 5 | 4 | 5 | | 4 | NA | NA | 5 | 5 | 5 | 5 |
| M355 | 6 | 6 | 6 | 6 | NA | | 6 | 5 | 4 | 5 | 5 | 4 | 4 |
| M357 | 4 | 6 | 4 | 5 | 5 | | NA | 4 | 6 | 2 | 4 | 2 | 2 |
| M36 | 6 | 5 | 5 | 5 | 5 | | 5 | 5 | 5 | 6 | 5 | 6 | 6 |
| M365 | 6 | 5 | 6 | 5 | 6 | | 5 | 6 | 5 | 4 | 5 | 4 | 6 |
| M373 | 5 | 4 | 6 | 5 | 5 | | 5 | 4 | 4 | NA | 5 | NA | 4 |
| M374 | 5 | 5 | 5 | 5 | 3 | | 6 | 5 | 6 | 4 | NA | 2 | 5 |
| M376 | 5 | 5 | 6 | 5 | 5 | | 6 | 4 | 4 | 6 | 6 | 5 | 5 |
| M382 | 6 | 5 | 5 | 5 | 5 | | 5 | 5 | 5 | 5 | 3 | 6 | 5 |
| M383 | 5 | 4 | 6 | 5 | 6 | | NA | NA | 6 | 5 | NA | 5 | 5 |
| M42 | 5 | 6 | 5 | 6 | 6 | | 5 | 4 | 5 | 6 | 6 | 4 | 4 |
| M52 | 4 | 3 | 5 | 5 | NA | | 5 | NA | 5 | 5 | 5 | 5 | 4 |
| M53 | 5 | 4 | NA | 5 | NA | | 4 | NA | NA | 6 | NA | 5 | 5 |
| M68 | 6 | 6 | 5 | 6 | 5 | | 4 | 4 | 6 | 6 | 3 | 5 | 3 |
| M78 | 5 | 6 | 5 | 5 | 6 | | 6 | 6 | 5 | 5 | 6 | 6 | 6 |
| M84 | 5 | 5 | 6 | 3 | 4 | | 5 | 6 | 6 | NA | 6 | 6 | 6 |
| M92 | 4 | 4 | 5 | 4 | 6 | | 5 | 5 | 5 | 5 | 5 | 6 | 5 |
| M95 | 5 | 5 | 5 | 5 | 6 | | 6 | 5 | 4 | 5 | 5 | 5 | NA |
| Statistical parameters | **Mean (SD)** | | | | | 5 (0.84) | | | | | | | |
|  | **Max** | | | | | 8 | | | | | | | |
|  | **Min** | | | | | 2 | | | | | | | |

**Table S2: Phenotypic data obtained for the panel of MAGIC lines.** For each line, transpiration rate measured at a vapor pressure deficit of 2.70 KPa (TR), linear or segmented trend transpiration rate (TR trend), the adjusted means of seminal root angle (SRA) measured in sexagesimal degrees were reported. Numbers between brackets point out the 1.96 standard deviations of each measurement. For each trait, maximum (Max), minimum (Min) average (Mean) values were calculated at the end of the table.

| Genotype | | TR ($\boldsymbol{mg}_{\boldsymbol{H}\boldsymbol{2}\boldsymbol{O}}\boldsymbol{m}^{\boldsymbol{-2}}\boldsymbol{s}^{\boldsymbol{-1}}$) | TR trend | SRA  (Sexagesimal degrees) |
| --- | --- | --- | --- | --- |
| Aldebaran | | 6.76 (1.61) | Linear | 78.25° (31.86°) |
| Athene | | 11.63 (2.29) | Linear | 71.88° (26.37°) |
| Dea | | 8.69 (0.22) | Segmented | 87.63° (27.87°) |
| Fridericus | | 7.51 (0.93) | Linear | 100.66° (32.46°) |
| Hatif de Grignon | | 7.36 (4.11) | Segmented | 70.83° (13.91°) |
| Ketos | | 5.04 (0.99) | Segmented | 82.35° (16.45°) |
| Ponente | | 9.44 (4.2) | Segmented | 87.45° (21.54°) |
| Robur | | 5.59 (0.27) | Linear | 88.5° (19.38°) |
| M101 | | NA | NA | NA |
| M102 | | NA | NA | NA |
| M106 | | 9.67 (1.6) | Segmented | 92.88° (29.84°) |
| M11 | | NA | NA | NA |
| M118 | | 5.98 (2.48) | Linear | 80.59° (15.79°) |
| M121 | | 8.59 (2.83) | Linear | 96.26° (13.66°) |
| M131 | | 8.27 (6.78) | Segmented | 84.74° (26.34°) |
| M132 | | 4.08 (0.51) | Segmented | 105.83° (13.71°) |
| M136 | | 6.13 (0.66) | Segmented | 104.03° (12.87°) |
| M140 | | 11.57 (0.26) | Linear | 98.69° (27.41°) |
| M147 | | 5.75 (2.07) | Segmented | 85.14° (28.89°) |
| M148 | | NA | NA | NA |
| M149 | | 4.63 (0.67) | Segmented | 86.39° (26.38°) |
| M150 | | 13.6 (2.36) | Segmented | 73.88° (24.52°) |
| M151 | | 7.82 (3.14) | Segmented | 105.06° (30.47°) |
| M154 | | 6.2 (1.12) | Segmented | 81.5° (18.1°) |
| M156 | | 9.8 (0.56) | Segmented | 76.17° (18.02°) |
| M158 | | 5.18 (1.43) | Linear | 80.93° (17.78°) |
| M162 | | 9.27 (4.37) | Segmented | 87.87° (30.9°) |
| M163 | | 5.98 (1.12) | Segmented | 80.86° (22.91°) |
| M169 | | 9.42 (1.8) | Linear | 96.84° (35.14°) |
| M177 | | 6.78 (1.32) | Segmented | 87.86° (28.1°) |
| M18 | | 6.54 (0.28) | Segmented | 57.75° (28.91°) |
| M180 | | 9.57 (1.7) | Segmented | 82.85° (18.33°) |
| M181 | | 7.66 (0.61) | Segmented | 75.95° (37.33°) |
| M190 | | 8.25 (1.82) | Segmented | 89.04° (19.21°) |
| M194 | | 5.53 (0.96) | Segmented | 88.43° (22.19°) |
| M200 | | 10.83 (1.98) | Segmented | 80.87° (32.99°) |
| M201 | | 7.85 (1.08) | Segmented | 88.72° (20.67°) |
| M215 | | NA | NA | 95.58° (31.11°) |
| M216 | | 6.79 (1.16) | Linear | 82.09° (41.83°) |
| M22 | | 6.3 (1.22) | Segmented | 80.81° (38.32°) |
| M224 | | 9.85 (3.14) | Linear | 79.39° (19.19°) |
| M233 | | 7.1 (3.5) | Segmented | 72.1° (19.02°) |
| M237 | | 5.38 (1.11) | Linear | 93.14° (25.78°) |
| M242 | | 6.22 (0.33) | Segmented | 86.99° (23.44°) |
| M246 | | 9.08 (4.49) | Linear | 78.82° (22.03°) |
| M253 | | 6.47 (1.35) | Segmented | 92.4° (28.79°) |
| M254 | | 7.64 (0.53) | Segmented | 86.13° (17.98°) |
| M258 | | 10.78 (0.54) | Segmented | 91.09° (26.07°) |
| M259 | | 9.2 (3.75) | Segmented | 83.3° (24.15°) |
| M262 | | 6.29 (1.05) | Segmented | 78.59° (24.21°) |
| M264 | | NA | NA | 88.27° (17.23°) |
| M278 | | 9.22 (6.03) | Segmented | 74.83° (13.68°) |
| M281 | | 5.72 (1.81) | Segmented | 72.82° (27.13°) |
| M285 | | 6.05 (0.35) | Linear | 87.22° (23.97°) |
| M287 | | 7.55 (2.29) | Segmented | 87.6° (22.25°) |
| M288 | | 7.22 (2.42) | Segmented | 104.5° (44.59°) |
| M289 | | 7.92 (0.25) | Segmented | 95.83° (12.55°) |
| M298 | | 6.48 (1.55) | Linear | 87.07° (25.29°) |
| M299 | | 8.4 (2.63) | Segmented | 88.31° (23.65°) |
| M30 | | 7.19 (0.44) | Segmented | 87.68° (30.76°) |
| M304 | | 5.44 (1.86) | Segmented | 90.19° (29.96°) |
| M313 | | 5.7 (1.27) | Segmented | 87.68° (22.78°) |
| M314 | | 9.51 (2.46) | Segmented | 105.32° (20.61°) |
| M321 | | 8.87 (2.29) | Linear | 91.55° (20.34°) |
| M324 | | 7.54 (2.53) | Segmented | 106.4° (31.21°) |
| M328 | | 7.29 (0.89) | Segmented | 77.79° (25.49°) |
| M332 | | 6.96 (2.12) | Segmented | 88.96° (15.21°) |
| M338 | | 8.52 (3.83) | Segmented | 80.58° (27.34°) |
| M339 | | 6.34 (2.66) | Segmented | 85.47° (18.24°) |
| M345 | | 6.14 (1.15) | Linear | 99.37° (22.49°) |
| M349 | | 8.09 (2.1) | Segmented | 77.41° (24.34°) |
| M350 | | 4.84 (0.23) | Linear | 92.29° (19.98°) |
| M355 | | 8.58 (1.75) | Segmented | 94.25° (17.99°) |
| M357 | | 6.67 (0.72) | Linear | 71.9° (32.73°) |
| M36 | | 7.17 (2.21) | Linear | 91° (20.5°) |
| M365 | | 8.66 (2.58) | Segmented | 92.65° (28.82°) |
| M373 | | 6.63 (3.37) | Segmented | 90.7° (29.11°) |
| M374 | | 4.91 (1.62) | Linear | 79.74° (29.34°) |
| M376 | | 9.75 (2.7) | Segmented | 85.51° (23.17°) |
| M382 | | 4.05 (0.96) | Linear | 89.87° (18.59°) |
| M383 | | 10.59 (5.17) | Linear | 87.06° (21.57°) |
| M42 | | 10.7 (2.1) | Linear | 85.5° (17.51°) |
| M52 | | 6.46 (2.04) | Linear | 78.55° (27.04°) |
| M53 | | NA | NA | 95.83° (16.36°) |
| M68 | | 7.89 (1.39) | Linear | 85.97° (25.48°) |
| M78 | | 8.11 (0.68) | Segmented | 90.99° (18.97°) |
| M84 | | 4.93 (0.2) | Linear | 74.27° (25.71°) |
| M92 | | 5.4 (1.4) | Linear | 85.33° (30.05°) |
| M95 | | 9.64 (4.71) | Segmented | 79.87° (27.96°) |
| Statistical parameters | **Mean** | 7.55  (1.91) | / | 86.61° |
|  | **Max** | 13.6 | / | 106.40° |
|  | **Min** | 4.05 | / | 57.75° |

**Table S3: Estimated parameters of the SRN-Models 1-5 used to predict SRN.** Posterior average (Mean) values along with the 1.96 standard deviation between brackets of fixed effects (*β_1_, β_2_,… β_12_*) were estimated.

| Model | β_1_ | β_2_ | β_3_ | β_4_ | β_5_ | β_6_ | β_7_ | β_8_ | β_9_ | β_10_ | β_11_ | β_12_ |
| --- | --- | --- | --- | --- | --- | --- | --- | --- | --- | --- | --- | --- |
| SRN-Model 1 | -0.90 (0.41) | -1.15 (0.42) | -0.96 (0.41) | -1.25 (0.43) | -1.30 (0.43) | -1.18 (0.43) | -1.30 (0.43) | -1.33 (0.43) | -1.32 (0.43) | -1.41 (0.43) | -1.53 (0.44) | -1.62 (0.44) |
| SRN-Model 2 | -0.68 (0.35) | -0.94 (0.36) | -0.75 (0.36) | -1.03 (0.37) | -1.08 (0.37) | -0.96 (0.37) | -1.08 (0.37) | -1.12 (0.38) | -1.10 (0.37) | -1.19 (0.37) | -1.32 (0.38) | -1.40 (0.38) |
| SRN-Model 3 | -0.88 (0.39) | -1.14 (0.40) | -0.95 (0.40) | -1.23 (0.41) | -1.28 (0.41) | -1.17 (0.41) | -1.28 (0.41) | -1.33 (0.41) | -1.31 (0.41) | -1.40 (0.41) | -1.52 (0.42) | -1.61 (0.42) |
| SRN-Model 4 | -0.93 (0.38) | -1.19 (0.39) | -1.00 (0.39) | -1.28 (0.40) | -1.33 (0.40) | -1.21 (0.40) | -1.33 (0.40) | -1.37 (0.41) | -1.36 (0.40) | -1.45 (0.40) | -1.57 (0.41) | -1.66 (0.41) |
| SRN-Model 5 | -0.81 (0.33) | -1.07 (0.34) | -0.88 (0.34) | -1.16 (0.35) | -1.21 (0.35) | -1.10 (0.35) | -1.22 (0.35) | -1.25 (0.35) | -1.24 (0.35) | -1.33 (0.35) | -1.45 (0.35) | -1.55 (0.36) |

**Table S4: Estimated thresholds of the SRN-Models 1-5.** Posterior average (Mean) values along with 1.96 standard deviations between brackets of threshold parameters ($\gamma_{1}, \gamma_{2},..\gamma_{6}$) were calculated.

| Model | $\boldsymbol{\gamma}$_1_ | $\boldsymbol{\gamma}$_2_ | $\boldsymbol{\gamma}$_3_ | $\boldsymbol{\gamma}$_4_ | $\boldsymbol{\gamma}$_5_ | $\boldsymbol{\gamma}$_6_ |
| --- | --- | --- | --- | --- | --- | --- |
| SRN-Model 1 | -3.77 (0.47) | -3.09 (0.46) | -2.11 (0.45) | -0.72 (0.41) | 1.13 (0.17) | 1.30 (0.10) |
| SRN-Model 2 | -3.52 (0.40) | -2.85 (0.39) | -1.88 (0.38) | -0.50 (0.36) | 1.21 (0.14) | 1.34 (0.07) |
| SRN-Model 3 | -3.76 (0.44) | -3.07 (0.43) | -2.10 (0.42) | -0.71 (0.39) | 1.13 (0.17) | 1.31 (0.10) |
| SRN-Model 4 | -3.81 (0.43) | -3.13 (0.42) | -2.15 (0.41) | -0.75 (0.39) | 1.11 (0.16) | 1.30 (0.10) |
| SRN-Model 5 | -3.70 (0.37) | -3.01 (0.35) | -2.03 (0.35) | -0.63 (0.33) | 1.17 (0.13) | 1.32 (0.08) |

**Table S5: Predictive ability of the SRN-Models 1-5:** Proportion of cases correctly classified (PCCC) using five TGBLUP models; Brier scores obtained from LOO cross-validation of the five TGBLUP models. For each model, maximum (Max), minimum (Min) average (Mean) values along with 1.96 standard deviations between brackets were calculated.

| Model | Prediction ability by Brier Score | | | Proportion of cases correctly classified (PCCC) | |
| --- | --- | --- | --- | --- | --- |
|  | Max | Mean | Min | Correctly predicted | Incorrectly predicted |
| SRN-Model 1 | 0.65 | 0.36 (0.08) | 0.21 | 451 (47.93%) | 490 (52.07%) |
| SRN-Model 2 | 0.65 | 0.36 (0.08) | 0.21 | 494 (47.50%) | 447 (52.50%) |
| SRN-Model 3 | 0.65 | 0.36 (0.08) | 0.21 | 491 (47.82%) | 450 (52.18%) |
| SRN-Model 4 | 0.65 | 0.36 (0.08) | 0.21 | 491 (47.82%) | 450 (52.18%) |
| SRN-Model 5 | 0.65 | 0.36 (0.08) | 0.21 | 490 (47.93%) | 451 (52.07%) |

**Table S6: Predictive ability and estimated variance components of the SRN-log Models 1-5:** The predictive ability points out the Pearson’s correlation between estimated and observed SRN using LOO cross-validation of the five extended GBLUP models; L indicates the estimated variance of line effects; G is the estimated variance of marker effects while GxG points out the variance of additive x additive epistatic effects. Numbers between brackets point out 1.96 standard deviations of the estimated variances.

| Model | Genomic heritability | Prediction ability | Estimated variance components | | | | |
| --- | --- | --- | --- | --- | --- | --- | --- |
|  |  |  | L | G | GxG | Error variance | Total Variance |
| SRN-log-Model 1 | 0.50 | 0.79 | 0.38 (0.12) |  |  | 0.37 (0.12) | 0.75 |
| SRN-log-Model 2 | 0.36 | 0.35 |  | 0.29 (0.09) |  | 0.51 (0.10) | 0.80 |
| SRN-log-Model 3 | 0.51 (0.23) | 0.53 |  | 0.19 (0.08) | 0.22 (0.09) | 0.39 (0.11) | 0.80 |
| SRN-log-Model 4 | 0.52 | 0.60 | 0.22 (0.09) | 0.19 (0.08) |  | 0.37 (0.11) | 0.78 |
| SRN-log-Model 5 | 0.58 (0.38) | 0.65 | 0.15 (0.07) | 0.15 (0.09) | 0.16 (0.07) | 0.33 (0.11) | 0.79 |

**Table S7: Predictive ability and estimated variance component of the SRA-Models 1-5:** The predictive ability points out the Pearson’s correlation between estimated and observed SRA using LOO cross validation strategy of the five extended GBLUP models; L indicates the estimated variance of line effects; G is the estimated variance of marker effects while GxG points out the variance of additive x additive epistatic effects. Numbers between brackets point out 1.96 standard deviations of the estimated variances.

| Model | Genomic heritability | Predictive ability | Estimated variance components | | | | |
| --- | --- | --- | --- | --- | --- | --- | --- |
|  |  |  | L | G | GxG | Error variance | Total Variance |
| SRA-Model 1 | 0.5 | 0.73 | 44.38 (14.74) |  |  | 44.34 (14.68) | 88.72 |
| SRA-Model 2 | 0.32 | 0.19 |  | 31.93 (11.80) |  | 66.76 (13.24) | 98.70 |
| SRA-Model 3 | 0.45 (0.2) | 0.37 |  | 20.27 (8.94) | 24.60 (11.33) | 54.35 (14.08) | 99.23 |
| SRA-Model 4 | 0.5 | 0.49 | 28.09 (12.07) | 20.13 (8.53) |  | 46.98 (14.12) | 95.22 |
| SRA-Model 5 | 0.46 (0.37) | 0.53 | 21.01 (10.47) | 14.87 (6.98) | 17.34 (8.81) | 44.14 (13.92) | 97.37 |

**Table S8: Predictive ability and estimated variance components of the TR-Models 1-5 used:** The predictive ability points out the Pearson’s correlation between estimated and observed TR using LOO cross-validation of the five extended GBLUP models; L indicates the estimated variance of line effects; G is the estimated variance of marker effects while GxG points out the variance of additive x additive epistatic effects. Numbers between brackets point out 1.96 standard deviations of the estimated variances.

| Model | Genomic heritability | Predictive ability | Estimated variance component | | | | |
| --- | --- | --- | --- | --- | --- | --- | --- |
|  |  |  | L | G | GxG | Error variance | Total Variance |
| TR-Model 1 | 0.49 | 0.96 | 0.31 (0.10) |  |  | 0.32 (0.11) | 0.63 |
| TR-Model 2 | 0.30 | 0.68 |  | 0.22 (0.08) |  | 0.49 (0.09) | 0.72 |
| TR-Model 3 | 0.41 (0.19) | 0.89 |  | 0.14 (0.06) | 0.16 (0.07) | 0.42 (0.10) | 0.73 |
| TR-Model 4 | 0.49 | 0.95 | 0.21 (0.09) | 0.13 (0.05) |  | 0.34 (0.10) | 0.69 |
| TR-Model 5 | 0.5 (0.35) | 0.95 | 0.15 (0.08) | 0.10 (0.05) | 0.11 (0.05) | 0.34 (0.10) | 0.71 |
