# Supplementary figures and images for: High accuracy of genome-enabled prediction of belowground and physiological traits in barley seedlings
Source: G3 (Bethesda). 2022 Jan 31;12(3):jkac022. doi: 10.1093/g3journal/jkac022 (PMC8895982; doi:10.1093/g3journal/jkac022)

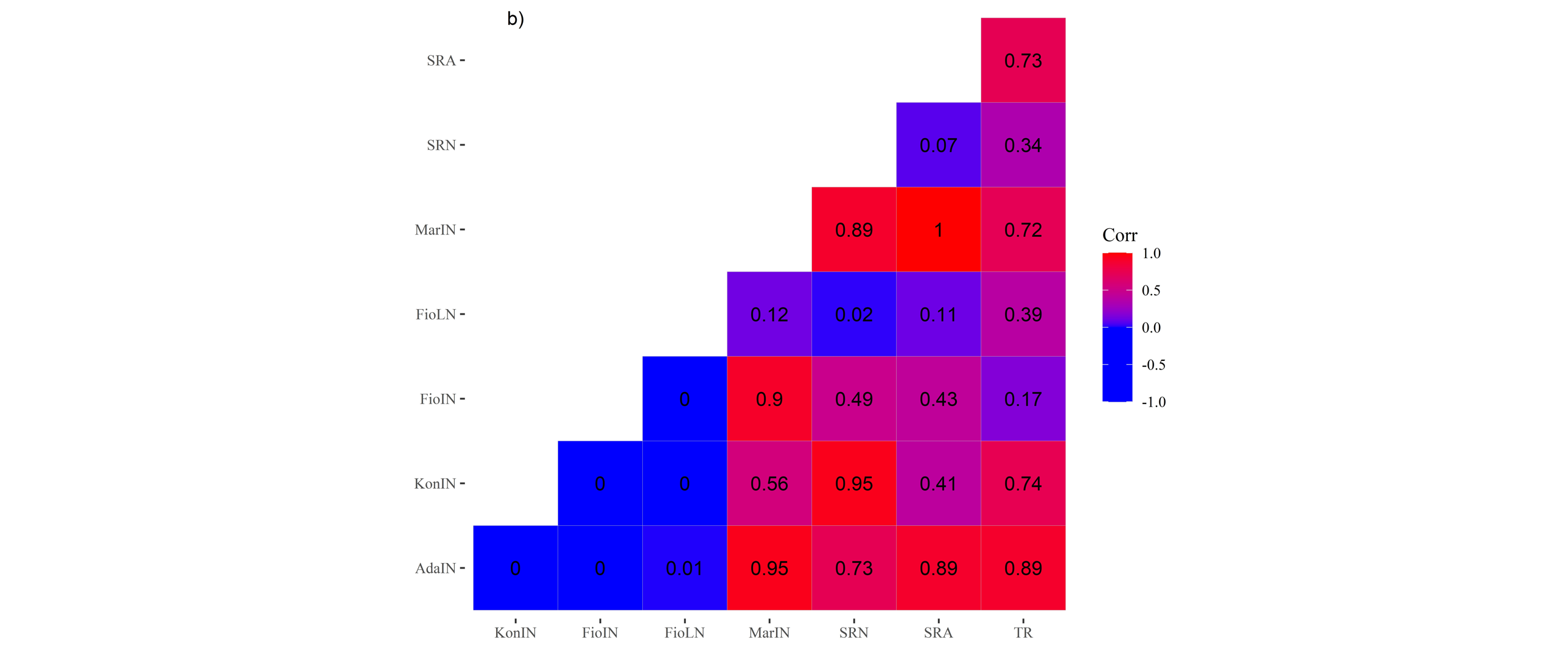

Supplement: jkac022_Figure_S1 [file jkac022_figure_s1.jpeg]
